# Supplementary material for: Lung structural cells are altered by influenza virus leading to rapid immune protection following re-challenge
Source: Nat Commun. 2025 Aug 1;16:7061. doi: 10.1038/s41467-025-62364-y (PMC12317152; doi:10.1038/s41467-025-62364-y)
Supplement: Supplementary file 1 — Supplementary Information [file 41467_2025_62364_MOESM1_ESM.pdf]

Supplementary Figure 1: Gating strategy for lung structural cells, sort purity for RNA sequencing, and PCA plots for day 10 and 40 post-infection

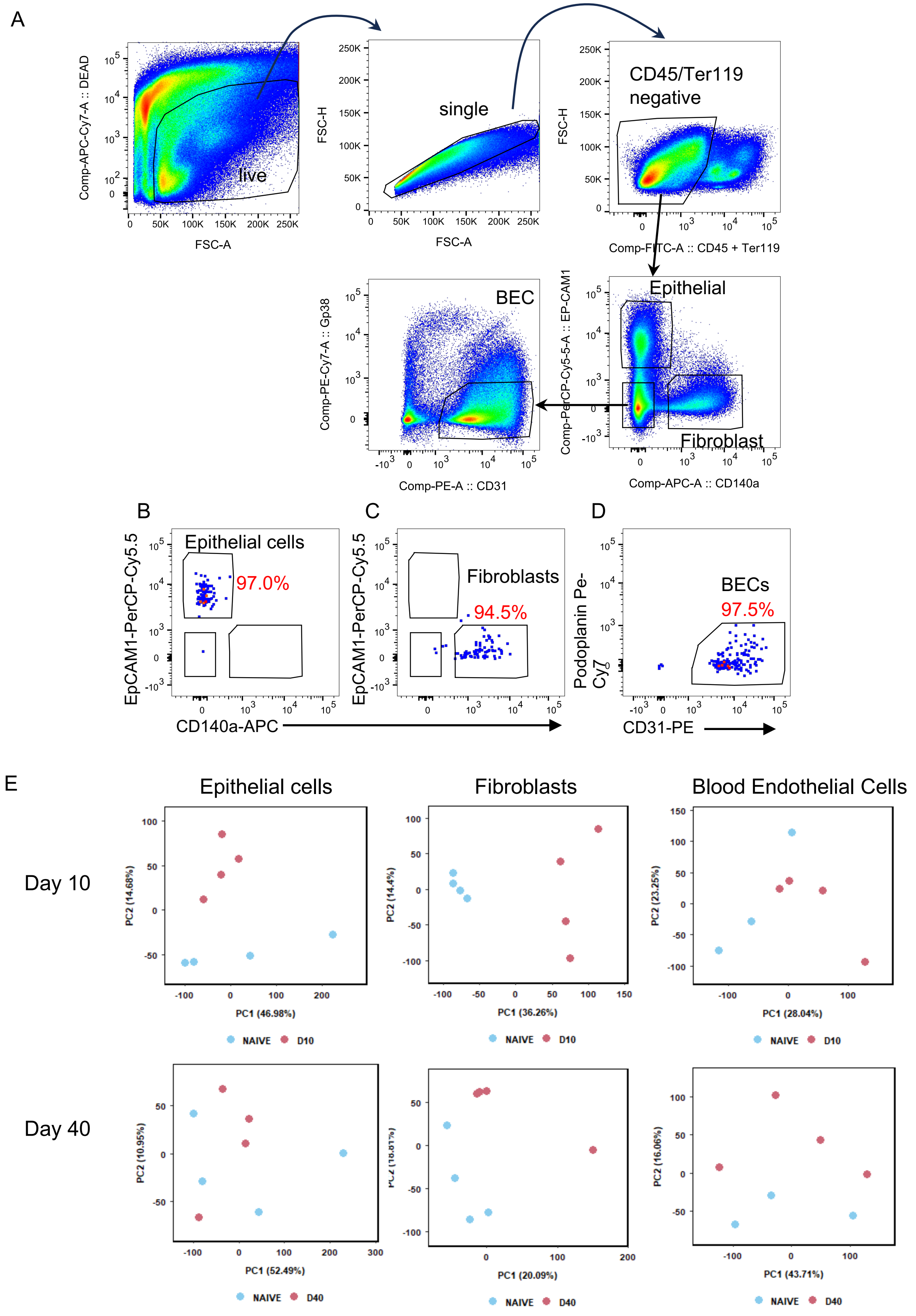

Supplementary Figure 1: Sorted structural gating strategy, sort purity for RNA sequencing and PCA plots for day 10 and 40 post-infection

A: Lung structural cells gated as indicated for FACS sorting. B: Post sort epithelial cells, C: post sort fibroblasts, D: post sort BECs. E: Principal Components Analysis (PCA) for day 10 and day 40 in which each component describes a proportion of the total underlying variation between genes and samples (ordered by the % of the underlying variation that they describe). Each point represents one mouse.

Supplementary Figure 2: ORA analysis of DEGs in lung structural cells 10 and 40 days following IAV infection

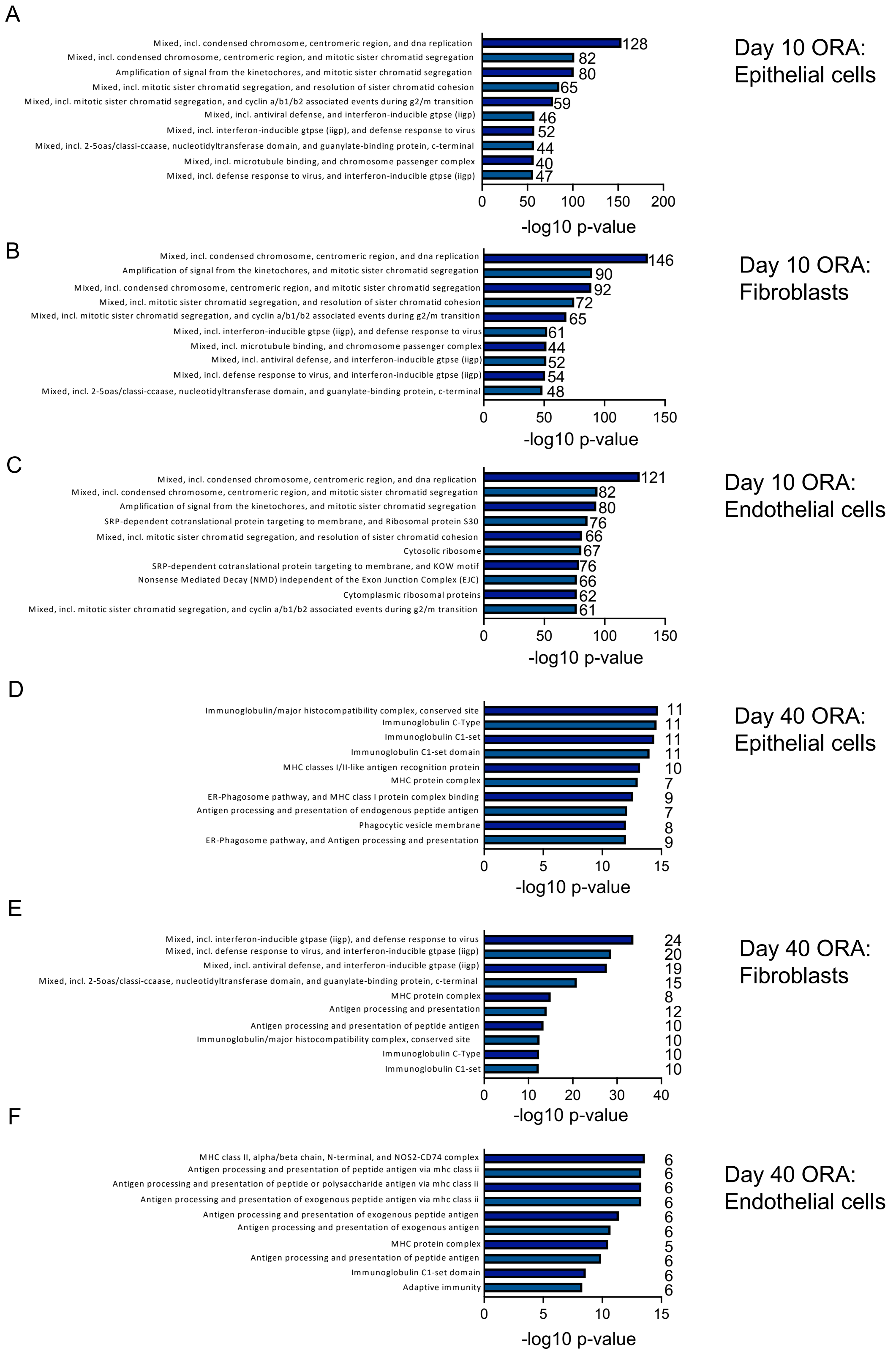

Supplementary Figure 2: ORA analysis of DEG in lung structural cells 10 and 40 days following IAV infection

RNA sequencing was performed on sorted lung epithelial cells, fibroblasts, and blood endothelial cells from naïve and C57BL/6 mice infected with IAV 10 (A-C) or 40 (D-F) days previously. Over Representation Analysis (ORA) bar charts showing ten most enriched gene-sets when using significantly upregulated genes with numbers next to each bar showing the number of genes in the pathway differentially expressed. To correct for multisampling a Benjamini-Hochberg correction was applied. Gene sets with an adjusted p-value of 0.05 and an absolute log2fold enrichment above 1 were considered significant. Exact adjusted p values are listed in the Source Data file.

Supplementary Figure 3: Correlation between RNA-seq and nCounter Analysis of epithelial and fibroblast gene expression following IAV infection

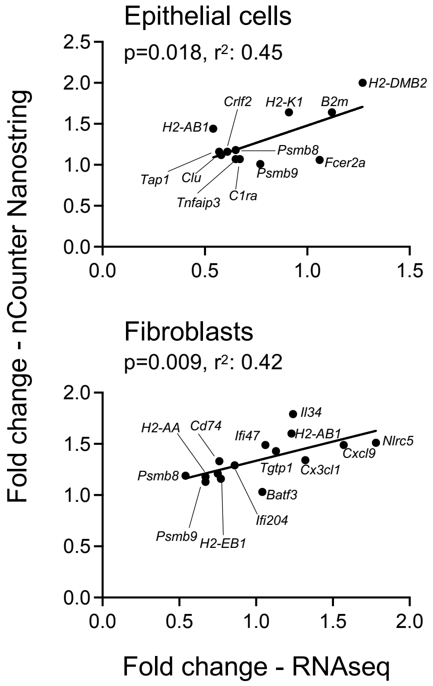

Supplementary Figure 3: Correlation between RNA-seq and nCounter Analysis of epithelial and fibroblast gene expression following IAV infection

C57BL/6 mice were either naïve or infected with IAV-WSN. 30-40 days later, lung epithelial cells and fibroblasts were FACS sorted. Gene expression was analyzed either by RNAseq or in a separate experiment by nCounter Nanostring analysis. Selected genes were shared between RNAseq DEG for each cell type and genes in the nCounter Nanostring mouse immunology panel. The data were analyzed using a Pearson correlation and lines on graphs shows the best fit based on linear regression analysis. Values are included in the Source Data file.

Supplementary Figure 4: Potential upstream regulators of DEG in lung structural cells post-IAV

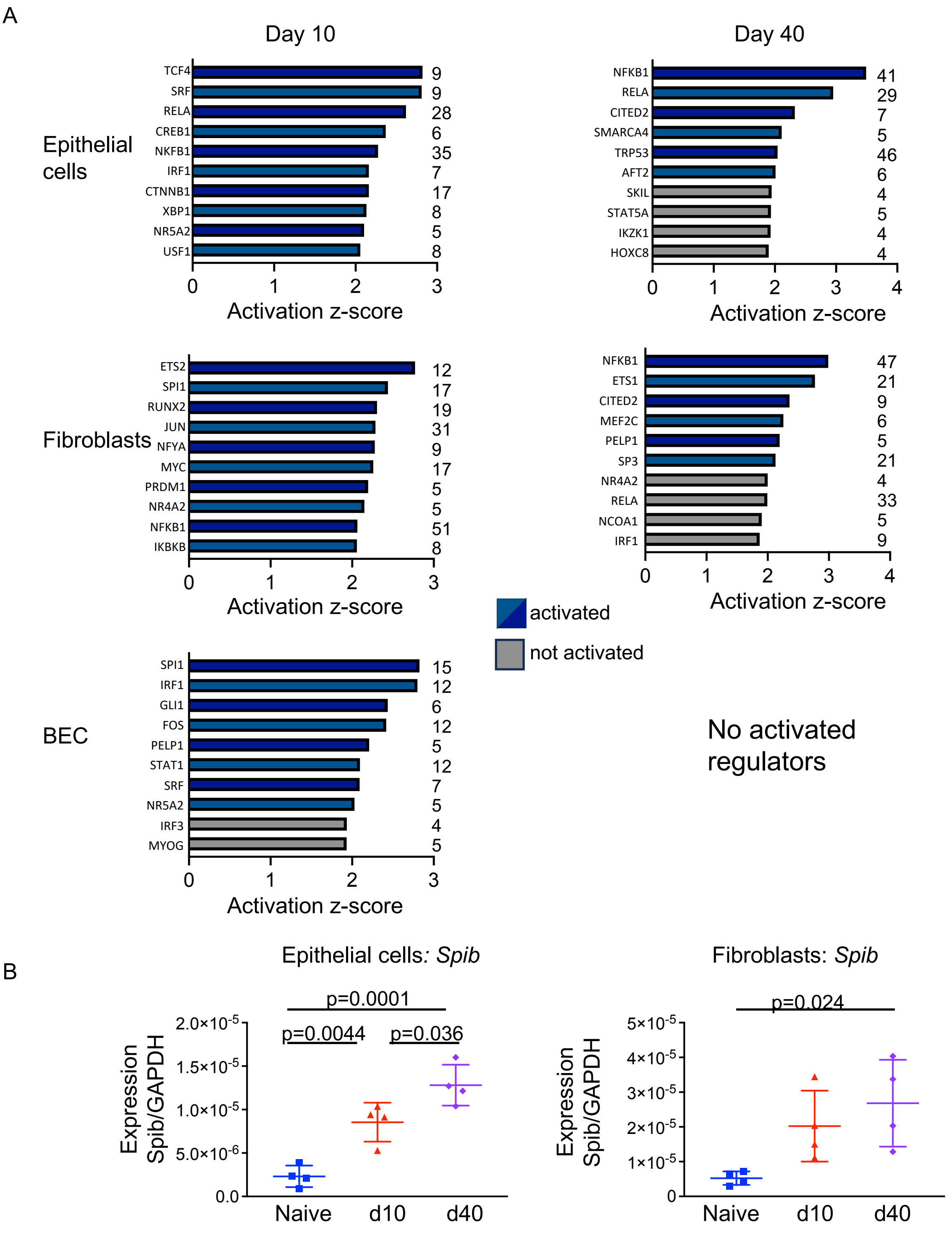

Supplementary Figure 4: Potential upstream regulators of DEG in lung structural cells post-IAV

A. RNA sequencing was performed on sorted lung epithelial cells, fibroblasts, and blood endothelial cells from naïve and C57BL/6 mice infected with IAV 10 and 40 days previously and potential upstream regulators identified by Activation z-score and the number at the end of each bar shows the number of genes activated in each pathway.
B. qPCR data on sorted cells showing absolute copy numbers of SpiB normalized to GAPDH in lung epithelial cells and fibroblasts. Data tested by an ANOVA followed by a Tukey's multiple comparison test. Each point represents a mouse and data are from one experiment.
Exact p values in A and values in B are included in the Source Data file.

Supplementary Figure 5: SpiB+ immune cells located in clusters are also B220 positive

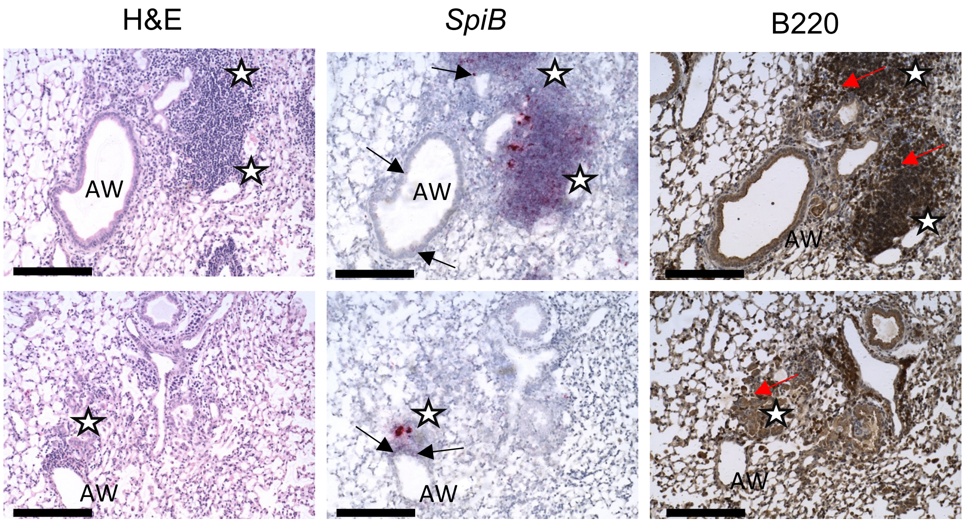

Supplementary Figure 5: SpiB+ immune cells located in clusters are also B220 positive

Immunohistochemistry and RNA-scope showing SpiB+ airway epithelial cells are located near B220+ immune cell clusters in the IAV infected lung at day 40 post IAV infection. Images taken at 20x magnification, scale bar 200µm. Airways (Aw), SpiB+ epithelial cells (black arrows), inflammatory foci (labelled with stars) and B220+ cells (red arrows).
Data are representative of 2 independent experiments. Expt 1: n=6, Expt 2: n=7 mice.

Supplementary Figure 6: Immune cell clusters do not form following infection with a replication deficient IAV virus (S-FLU)

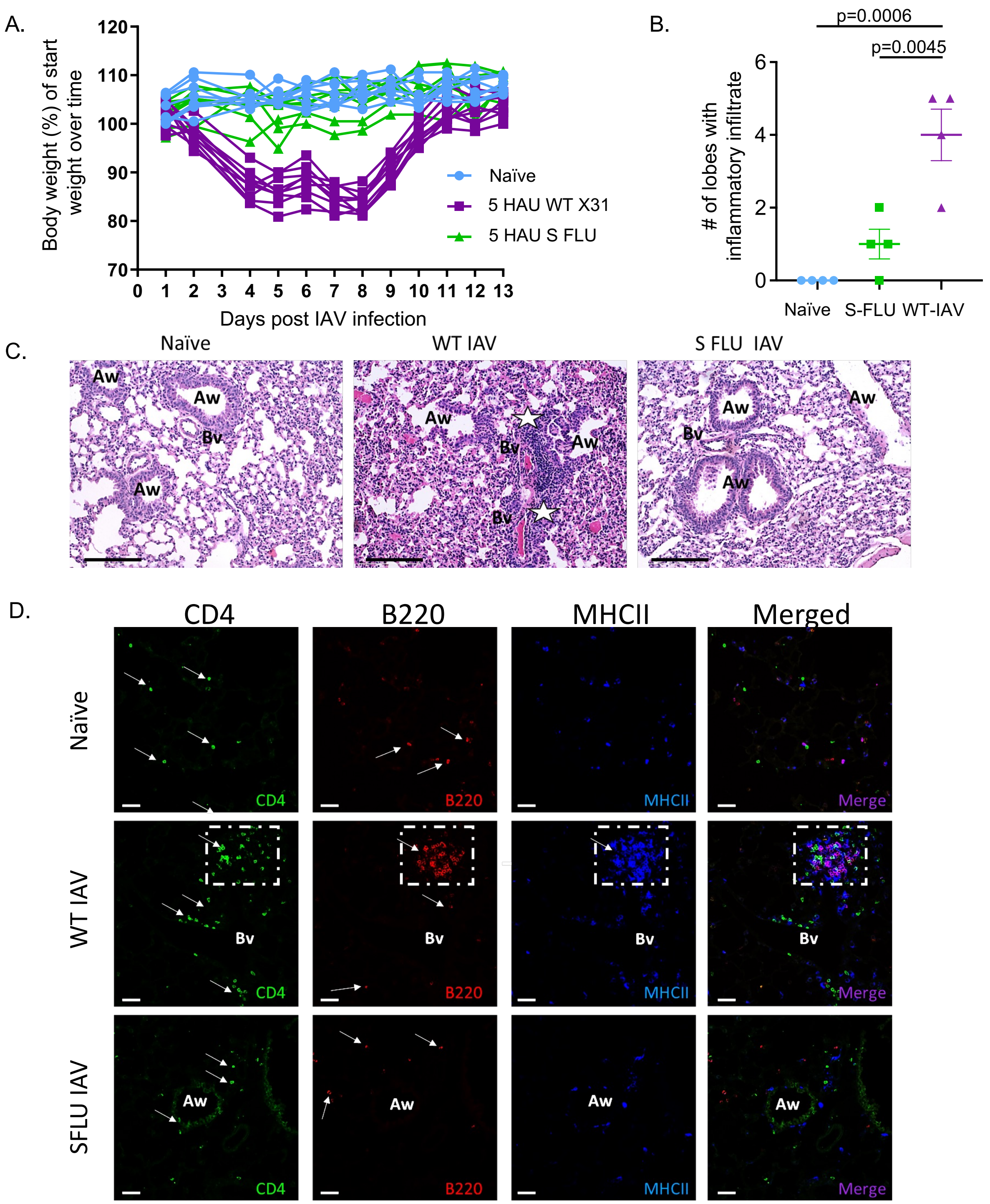

Supplementary Figure 6: Immune cell clusters do not form following infection with a replication deficient IAV virus (S-FLU)

C57BL/6 mice were infected i.n with IAV (X31 strain, either wild type or single cycle (S-FLU)). A: Weight loss graph showing percentage of total body weight over time post infection (n=9) mice per group (naïve, WT-IAV and S-FLU). Data are combined from two independent experiments. B: Histological analysis of inflammation in naïve, WT-IAV and S-FLU infected mice at day 30 post-infection. Data are normally distributed and shown as mean +/- SEM and tested by a one way-ANOVA with Šidák's multiple comparison. C: H&E staining showing airways (Aw), blood vessels (Bv) inflammatory foci/immune cell clusters (labelled with white stars) in naïve mice, and mice infected with WT-IAV and S-FLU 30 days previously. All images taken at 200x magnification, scale bar 200µm. D: Immunofluorescent staining shows the localization of CD4 T cells (green), B cells (B220+, red) and antigen presenting cells (MHCII, blue) in lung sections from naïve and IAV infected mice culled at day 30 post infection. All images taken at 200x magnification, scale bar 200µm. Airways (Aw), blood vessels (Bv) inflammatory foci/immune cell clusters (labelled with dashed white box). Positive cells are indicated by white arrows. Values in A and B are included in the Source Data file.

Supplementary Figure 7A: B cells and plasmacytoid DCs express the mCherry reporter molecule

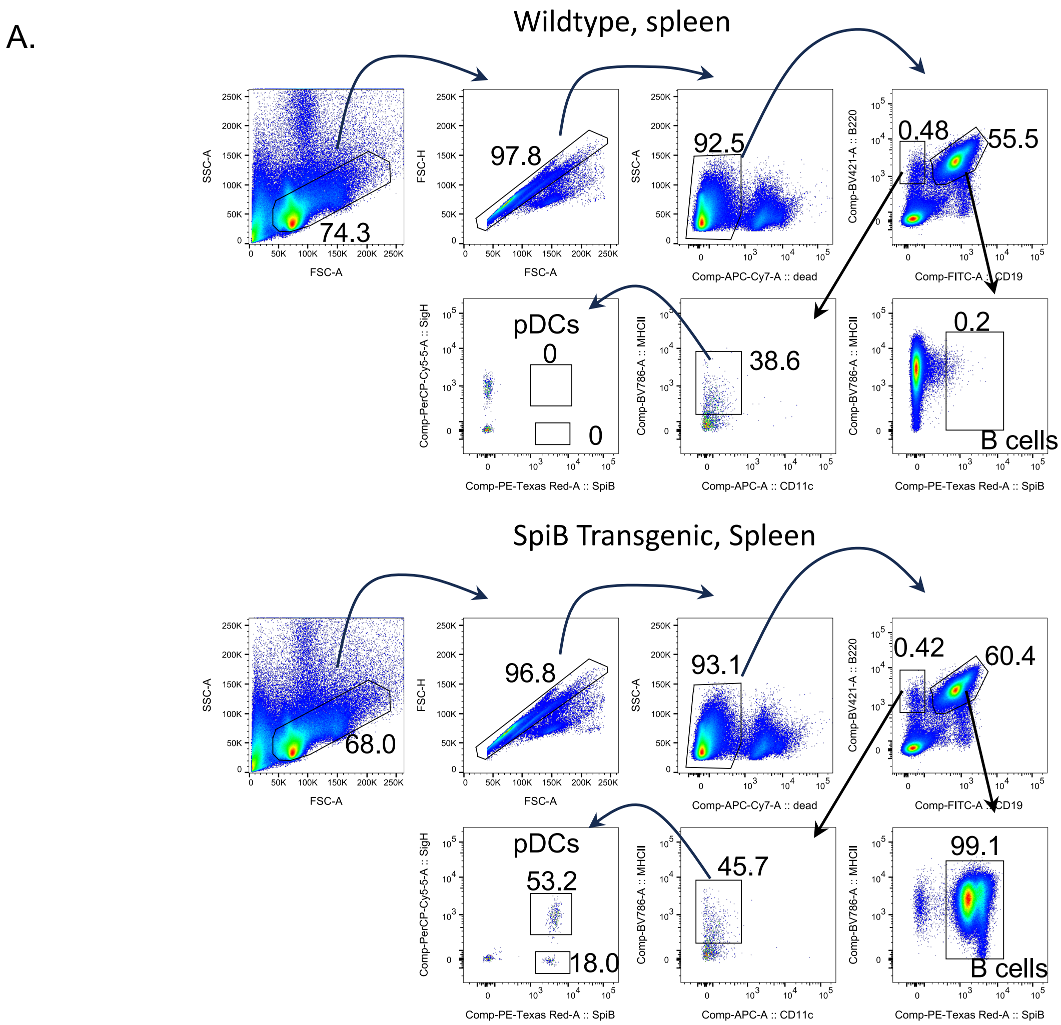

Supplementary Figure 7B: mCherry+ epithelial cells express *Spib* mRNA

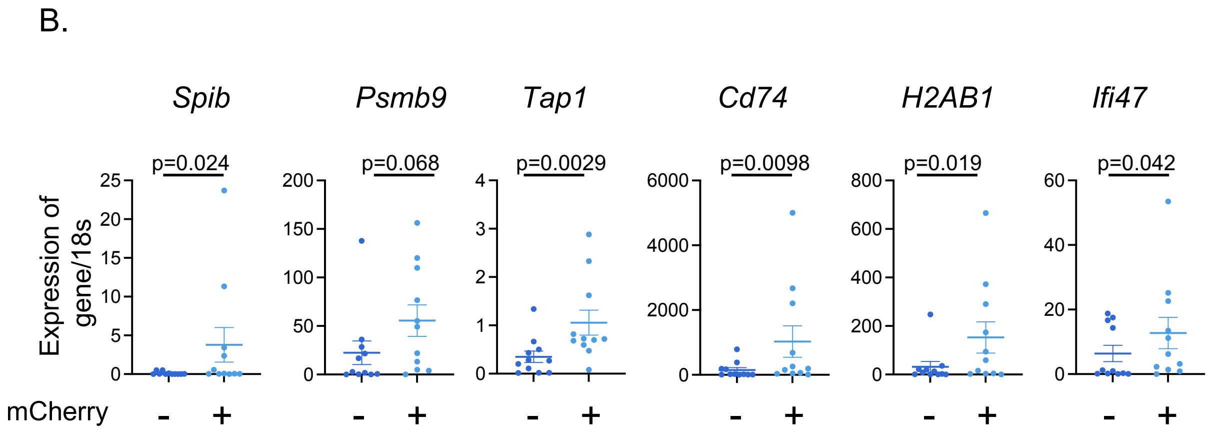

Supp Figure 7C: SpiB reporter validation and gating strategy for lung epithelial cell subsets

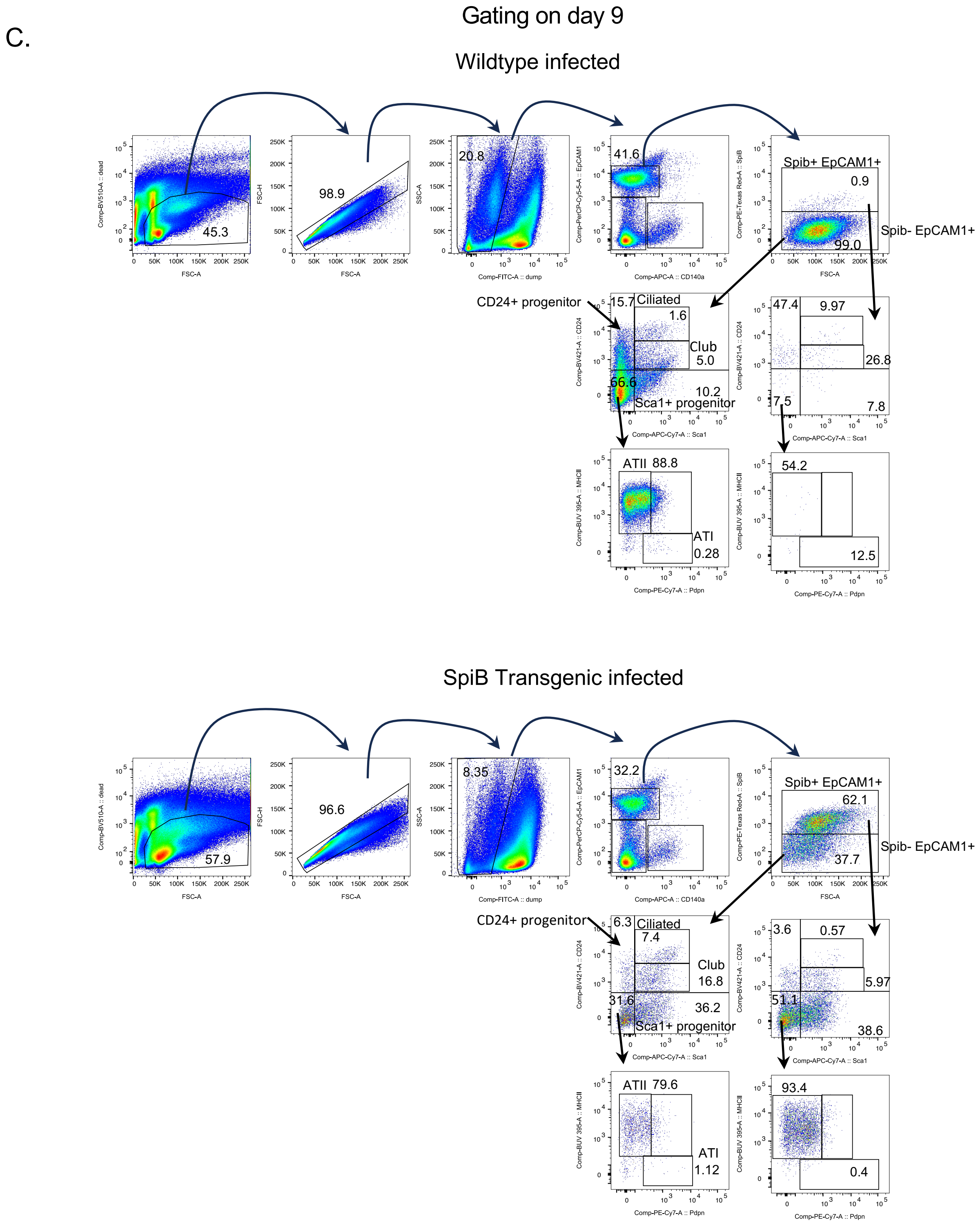

Supplementary Figure 7: SpiB reporter validation and gating strategy for lung epithelial cell subsets

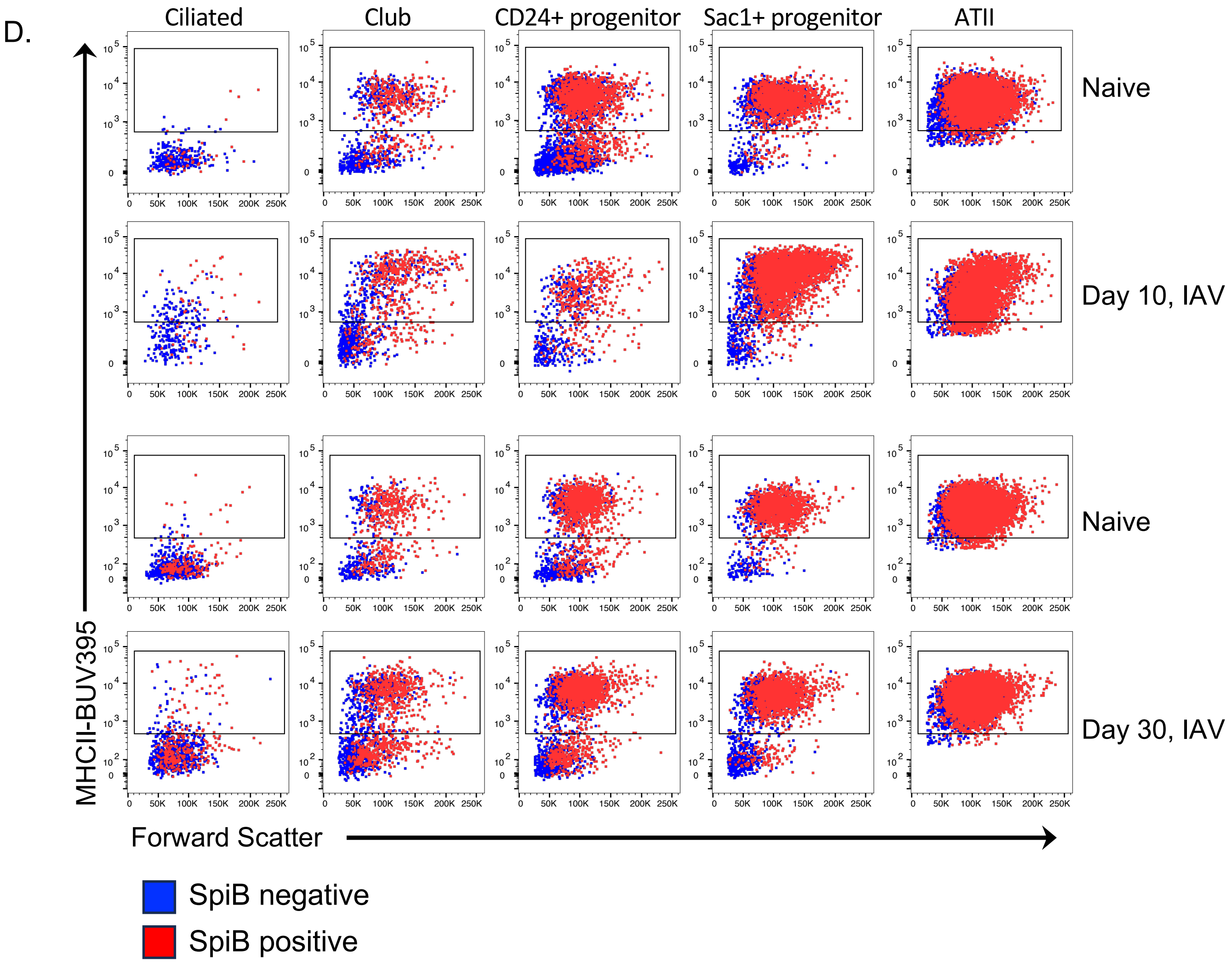

Supplementary Figure 7: SpiB reporter validation and gating strategy for lung epithelial cell subsets

A: Spleen cells from naïve SpiB-mCherry reporter mice and wildtype C57BL/6 mice were analysed by flow cytometry and B cells and plasmacytoid DCs identified. Numbers indicate percentages within the indicated gates.

B: SpiB-mCherry positive and negative cells were FACS sorted based on SpiB+ and SpiB negative EpCAM1+ cells based on the initial gating strategy described in C. cDNA was prepared from isolated RNA and examined by semi-quantitative PCR and normalised using the house keeping gene, 18s. Each point represents one mouse and data are combined from three separate sorts. Data are not normally distributed and tested via Wilcoxon tests. Values are included in the Source Data file.

C: SpiB-mCherry reporter mice and transgenic negative (Tg neg) littermates were infected i.n. with IAV (WSN) on day 0 and culled at day 9 post infection. Lung cells were gated on live, single, lineage negative (CD45/31, CD140a), EpCAM1+ epithelial cells that were either mCherry (SpiB) positive or mCherry (SpiB) negative. Subsets were then further identified: Ciliated cells (CD24hiSca1hi), club cells (CD24+Sca1+), CD24+ progenitors (CD24+Sca1-) Sca1+ progenitors (Sca1+CD24-), AT1 (CD24-Sca1- PDPN+) and ATII (CD24-Sca1-PDPN-MHCII+). Representative FACS plots are shown for (B) Tg negative littermates and (B) SpiB reporter mice. Numbers indicate the percentage within each gate.

D: Representative plots of the SpiB-mCherry negative (blue) and positive (red) epithelial cell populations showing MHCII expression, percentages MHCII positive cells graphed in Figure 4.

SpiB reporter flow cytometry data are representative of two independent experiments per time point with =11 naïve, N=7 primary and N=10 memory.

Supplementary Figure 8: MHCI expression is increased after IAV infection

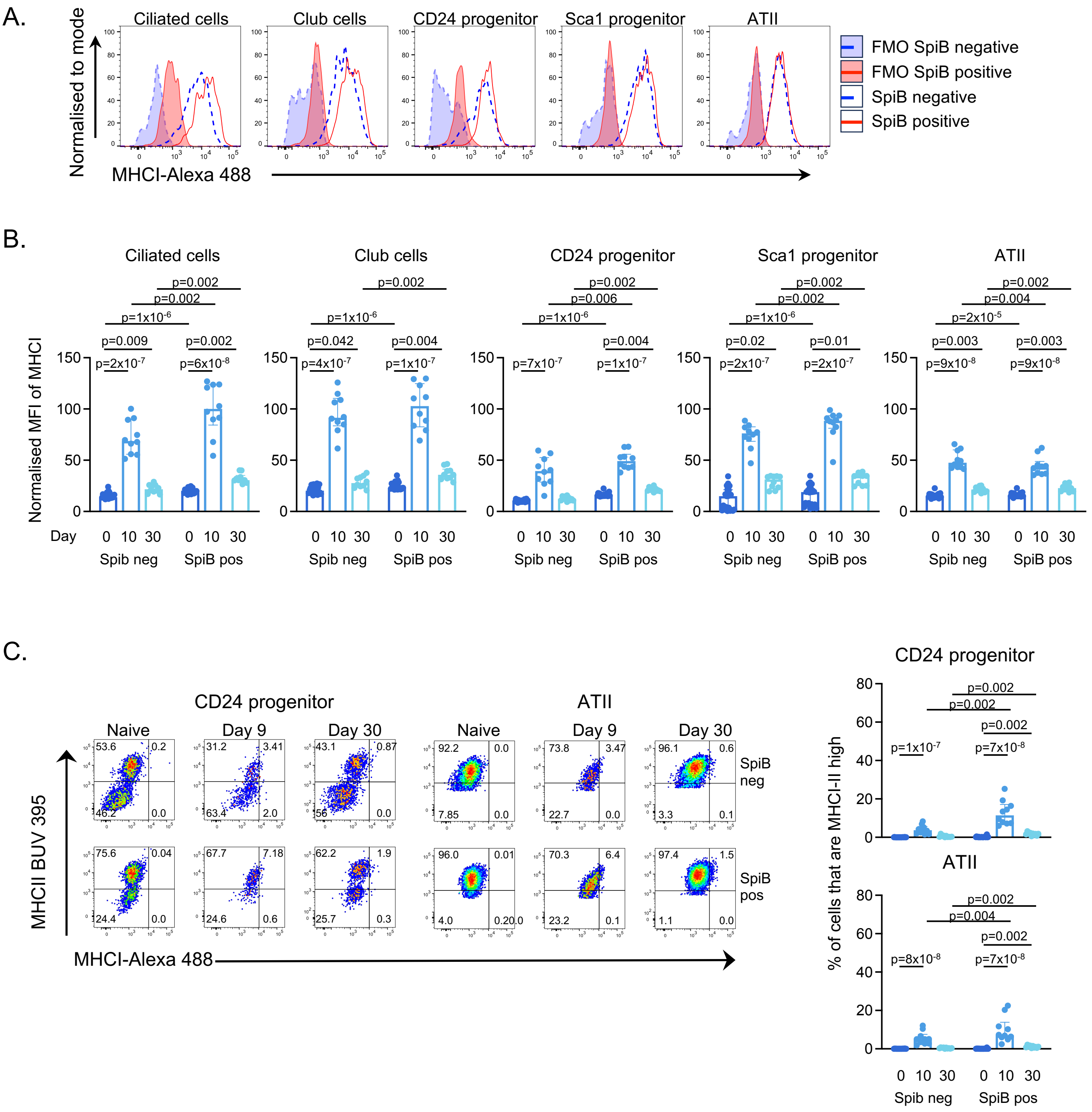

Supplementary Figure 8: Epithelial cell MHCI expression is increased after IAV infection

SpiB-mCherry reporter mice were infected with IAV (100-200PFU) and lungs taken at day 0, 10 or 30 post infection. EpCAM1+ epithelial cell populations were identified by gating on lineage (CD45, CD31 and CD140a) negative, SpiB negative or SpiB+ populations. A shows representative staining of MHCI on SpiB negative and positive epithelial cell populations from naïve mice and data are graphed in B. C shows representative staining of MHCI-MHCII high CD24 progenitor and ATII cells at each timepoint.

In the graphs each symbol represents a mouse and the bars show median with interquartile range as data are not normally distributed. Differences between time points were tested using a Kruskal-Wallis test followed by a Dunn's multiple comparisons test and between SpiB negative and positive populations by paired Wilcoxon rank tests.

All data are from two independent experiments with a total of n=21 naïve; n=10 day 10 IAV; n=10 day 30 IAV mice.

Values in B and D are included in the Source Data file.

Supplementary Figure 9: Ex vivo fibroblasts and gating strategy for identification of interferon responsive fibroblasts

A.

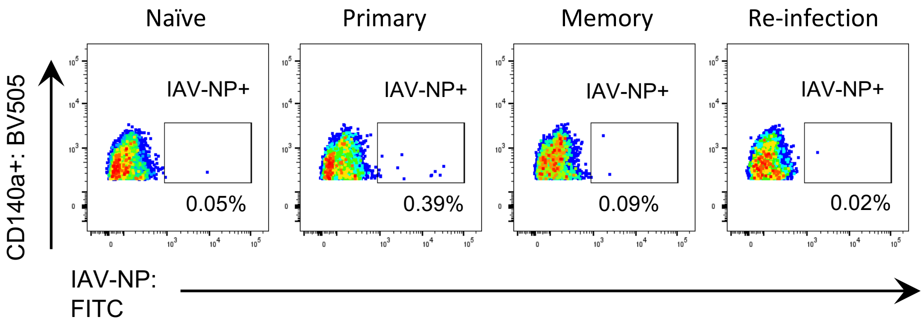

Supplementary Figure 9A: IAV-Nucleoprotein positive fibroblasts are only detected at day 2 post IAV challenge

C57BL/6 mice that were either naïve or infected 30 days earlier with IAV-WSN, were infected with IAV-X31, their lungs harvested 2 days later, and the presence of IAV-NP within the indicated CD140a+ population examined by flow cytometry. Cells are gated as shown in Supplementary Fig 1A. Data are representative of two experiments with a combined number of (n=8) for X31 primary and (n=9) for re-infected groups.

B.

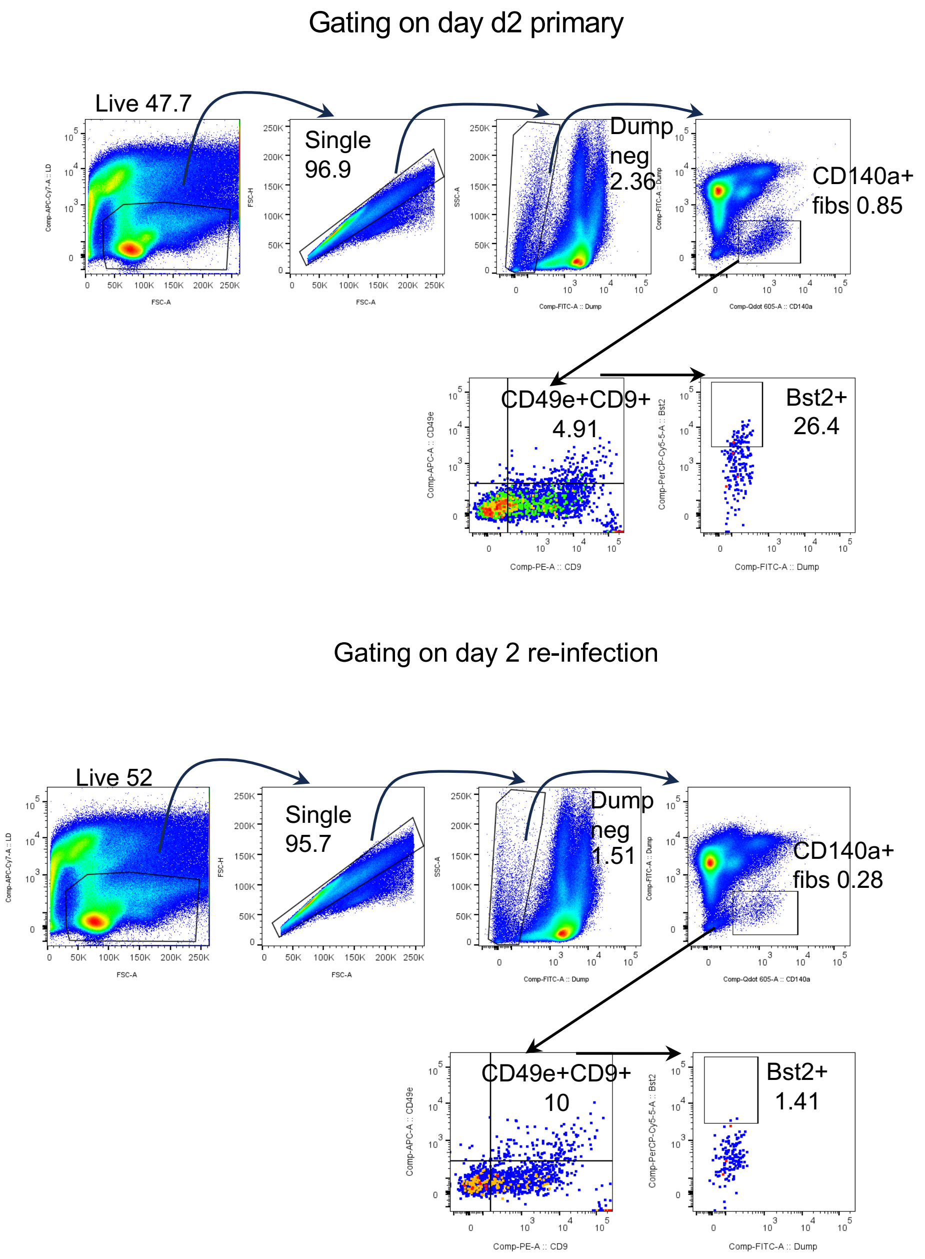

Supplementary Figure 9B: Gating strategy for identification interferon responsive fibroblasts

C57BL/6 mice that were either naïve or infected 30 days earlier with IAV-WSN, were infected with IAV-X31, their lungs harvested 2 days later, and the presence IFN responsive fibroblast populations was examined by flow cytometry. Cells are gated as shown in (A) primary d2 and (B) re-infected d2. Lung cells were gated on live, single, lineage negative (CD45/31/EpCAM1 negative), CD140a+ fibroblasts that were CD49e+CD9+ and then further gated on Bst2+ versus dump negative populations.

Supplementary Figure 10A: Gating to identify IAV-NP+ CD45+ lung cells at day 2 post-infection

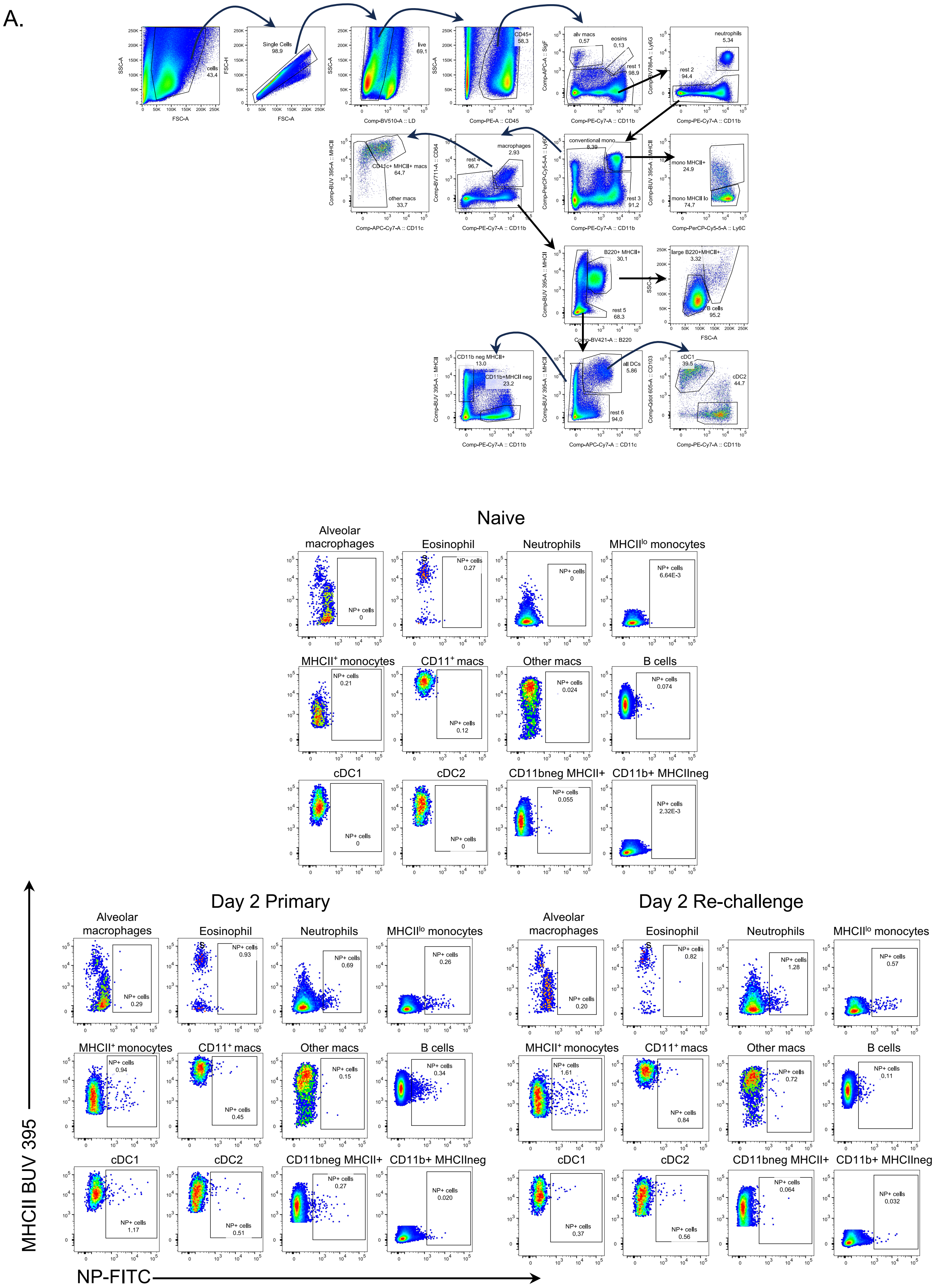

Supplementary Figure 10: Reduction in viral titers is not due to increased uptake of IAV-NP antigen by APC after IAV re-challenge

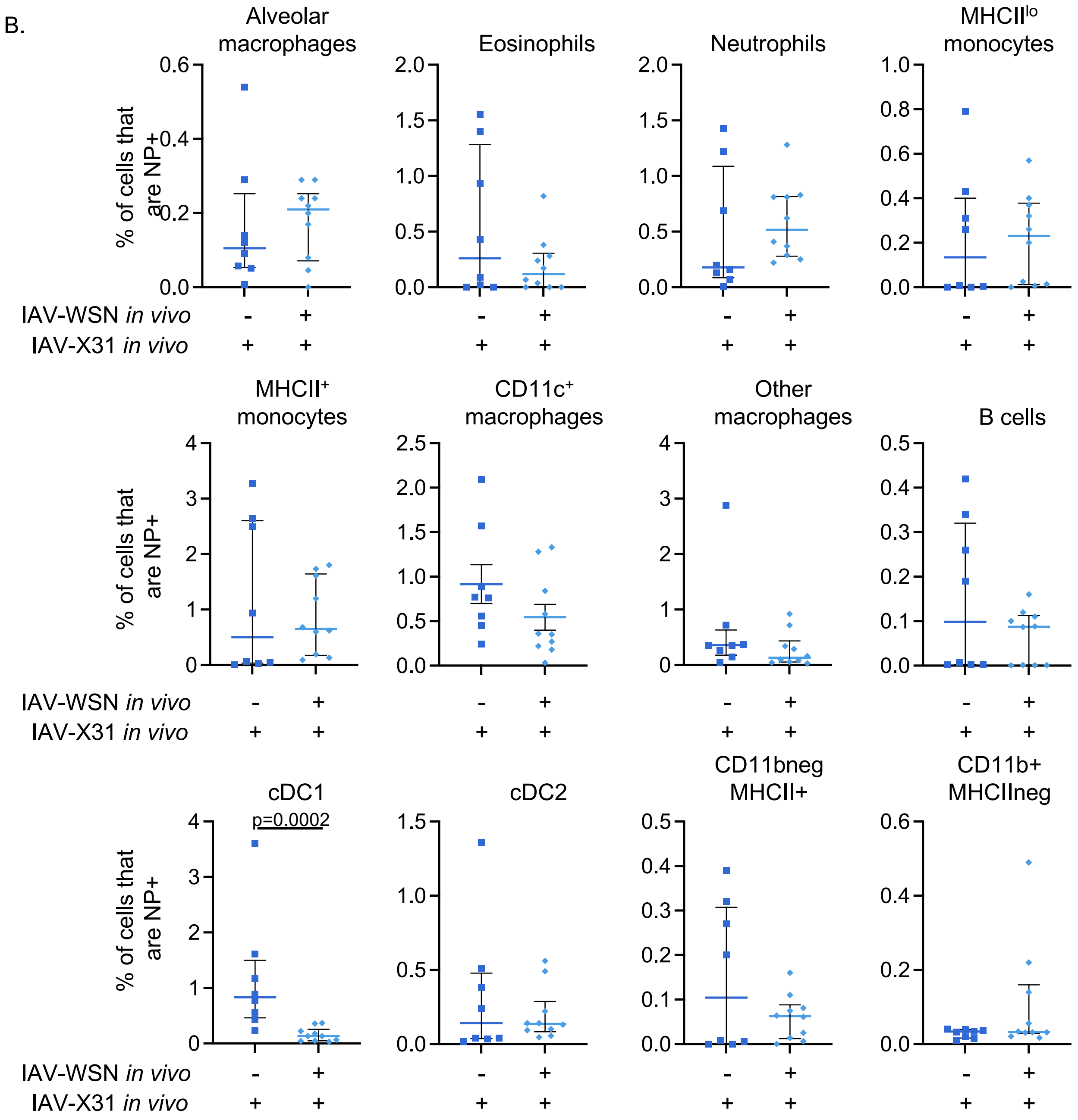

Supplementary Figure 10: Reduction in viral titers is not due to increased uptake of IAV-NP antigen by APC after IAV re-challenge

C57BL/6 mice that were either naïve or infected 30 days earlier with IAV-WSN, were infected with IAV-X31, their lungs harvested 2 days later, and the presence of IAV-NP within the indicated CD45+ populations examined by flow cytometry. Cells are gated as shown in (A) and combined data shown in (B). Data are from two experiments with a combined number of (n=8) for X31 primary and (n=9) for re-infected groups with each symbol representing a mouse and the horizontal line showing the median for all apart from CD11c+ macrophages where the line shows the mean, error bars are interquartile range expect from CD11c+ macrophages in which they show the SEM. All data apart from CD11c+ macrophage are not normally distributed and differences tested by Mann Whitney, expect from CD11c+ macrophages in which differences tested by a T-test.
Values in B are included in the Source Data file.

Supplementary Figure 11: Primary and re-infected mice have a type I IFN response signature in virus+ airways

A.

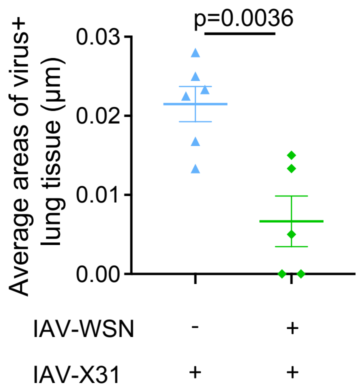

B.

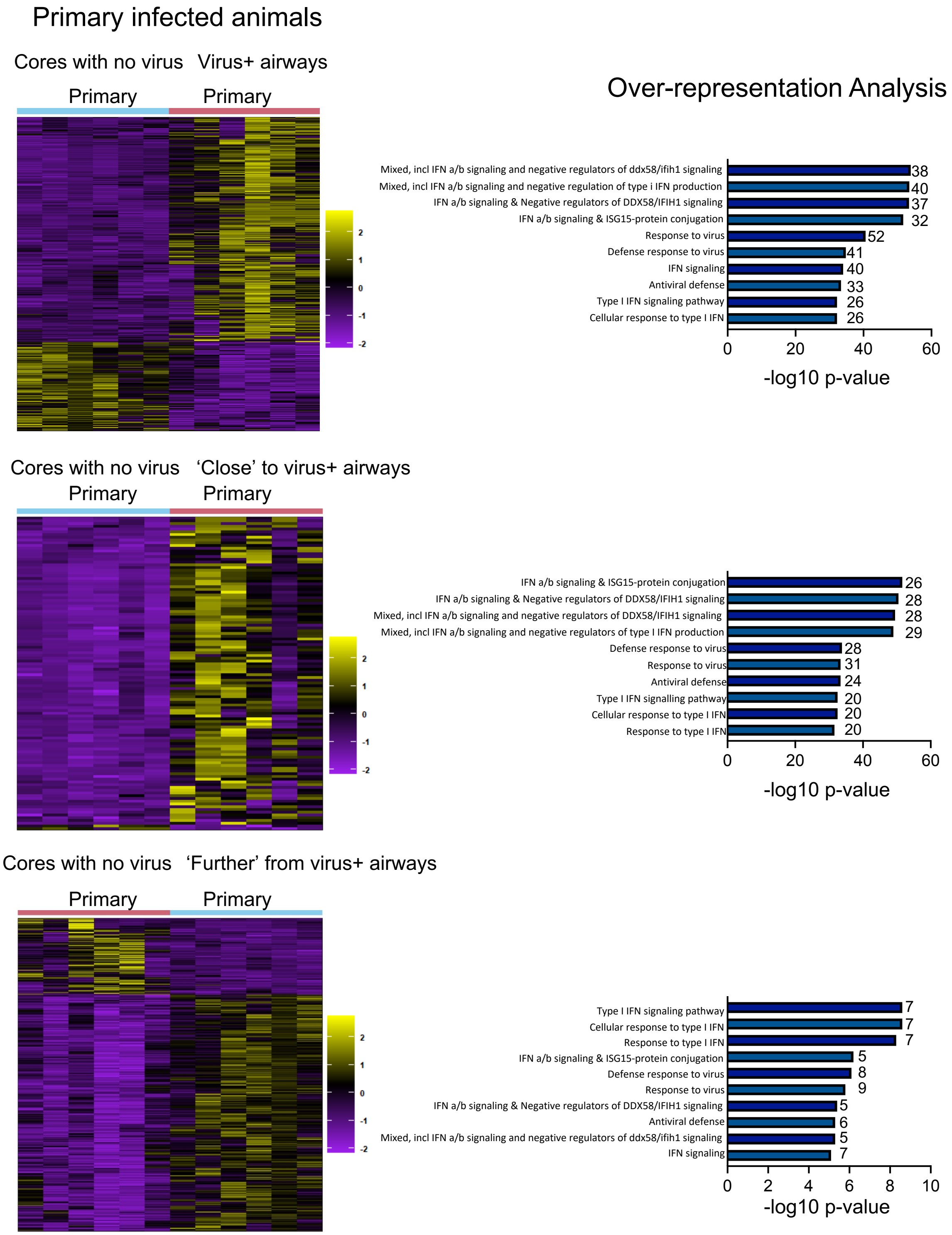

Supplementary Figure 11: Primary and re-infected mice have a type I IFN response signature in virus+ airways

C.

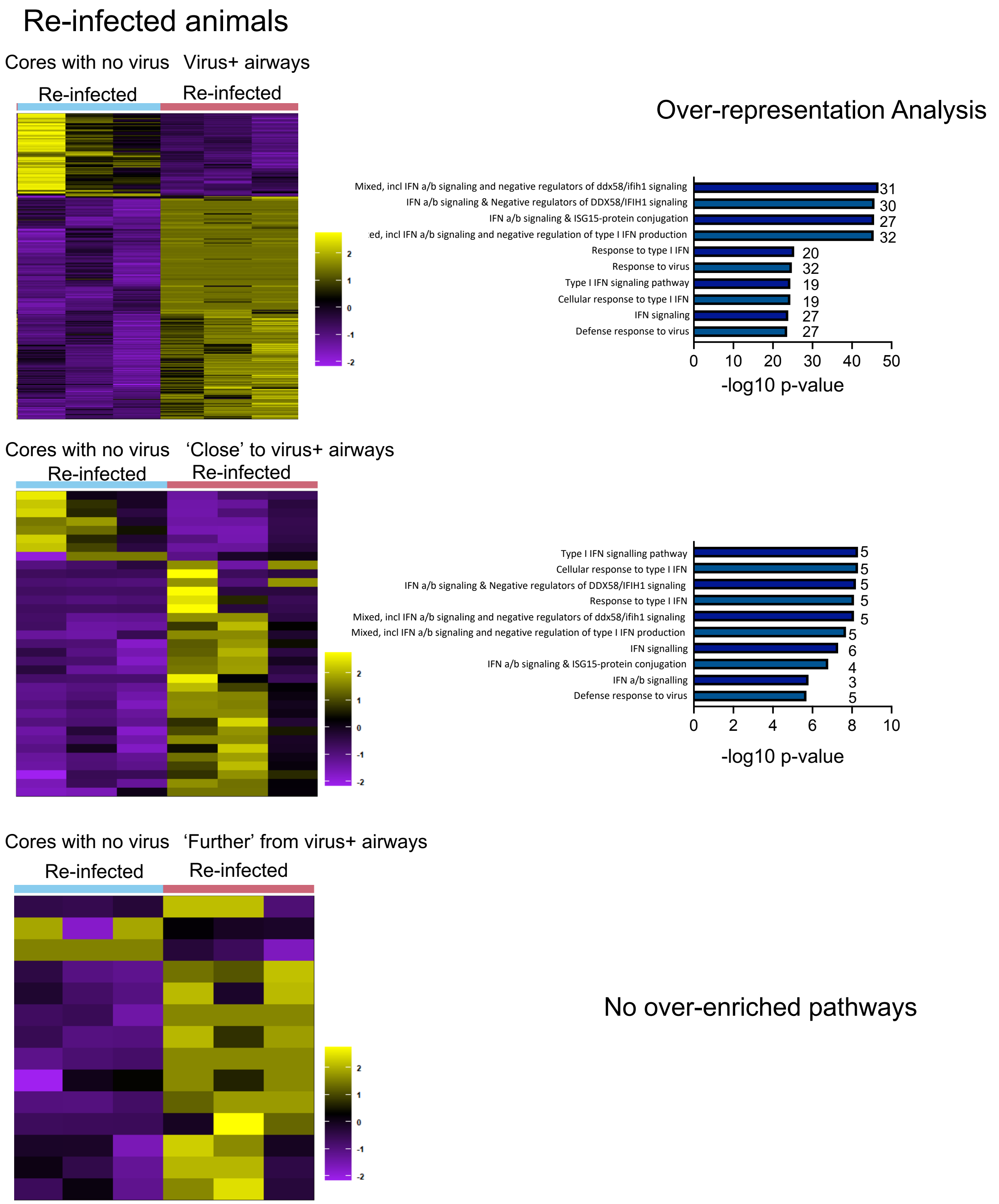

Supplementary Figure 11: Primary and re-infected mice have a type I IFN response signature in virus+ airways

C57BL/6 mice that were either naïve or infected 30 days earlier with IAV-WSN, were infected with IAV-X31, their lungs harvested 2 days later and embedded in paraffin. Virus positive and negative airways from one experiment with 6 primary and 3 re-infected mice were identified by RNAscope (A) and analysed by GeoMX. Heatmaps show DEG and enriched pathways in comparisons within primary (B) or re-infected (C) animals between airways from virus negative cores and either: virus+ airways; virus negative areas within the same airway as virus+ cells (Close); or airways adjacent to virus+ airways (Further). Over Representation Analysis (ORA) bar charts showing ten most enriched gene-sets when using significantly upregulated genes with numbers next to each bar showing the number of genes in the pathway differentially expressed.
Data in A and p values in B-C are included in the Source Data file.

Supplementary Figure 12: CD4 and CD8 T cells are depleted following two rounds of antibody treatment

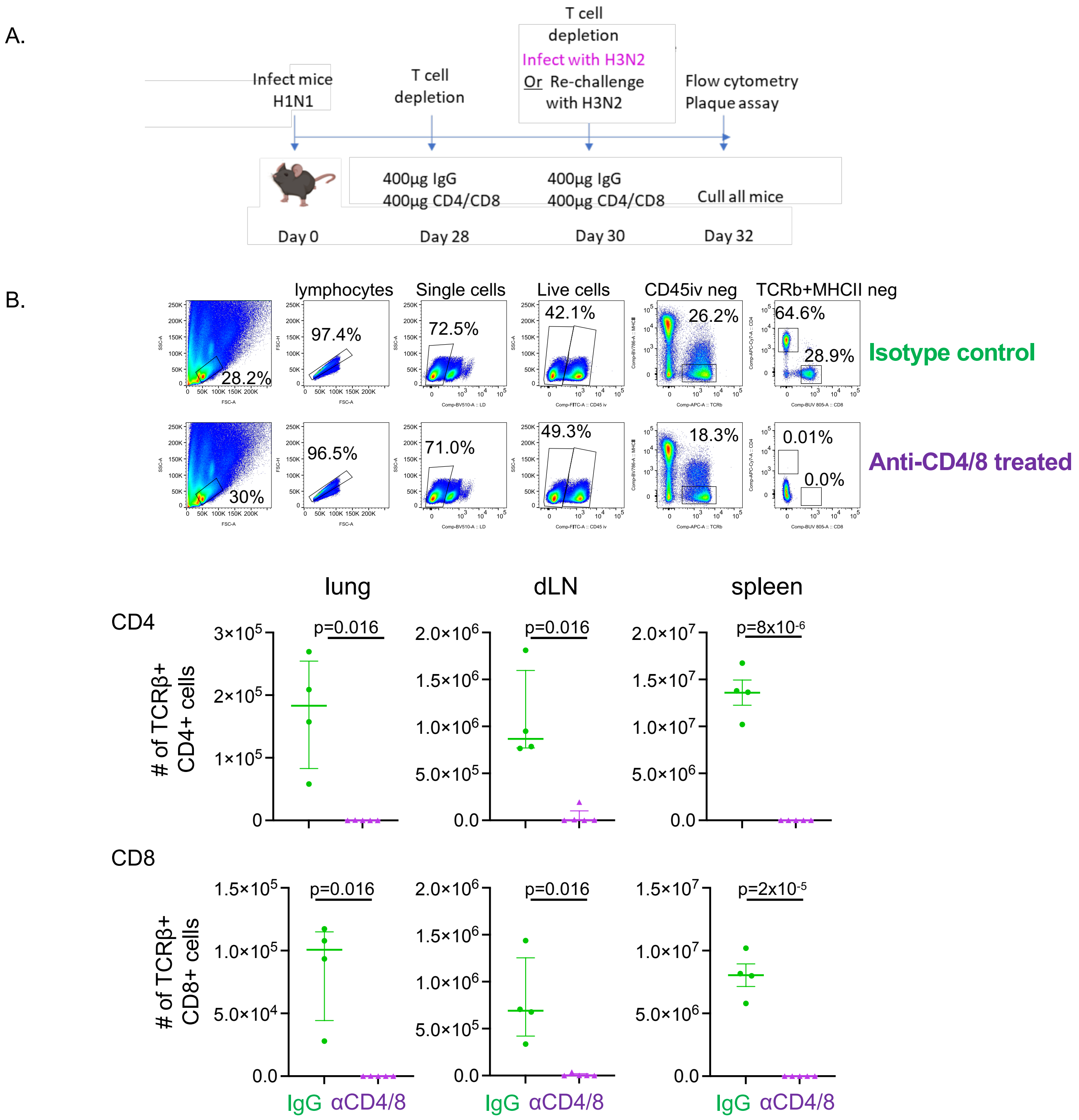

Supplementary Figure 12: CD4 and CD8 T cells are depleted following two rounds of antibody treatment

(A) Schematic created in BioRender (Created in BioRender. Worrell, J. (2025) https://BioRender.com/ux9t49r) displays experimental design: T cell depletion experimental timeline, C57BL/6 mice were infected with IAV-WSN on day 0 and treated with 400µg isotype control or 200µg each of anti-CD4 (GK1.5) and anti-CD8 (2.43) on days 28 and day 30 when mice were (re)-infected with IAV-X31. (B) Representative FACS plots showing CD4 and CD8 T cell depletion in the lung day 2 post infection, gated on live, single, CD45 iv negative lymphocytes, that are MHCII negative and TCRβ+ and numbers of CD4 and CD8 T cells that are TCRβ+ in isotype control (IgG) and T cell deleted groups at day 32. Cell numbers from the lung, draining lymph node and spleen in IgG treated group compared to anti-CD4 and CD8 treated groups and compared by a T test, apart from CD8 T cells in lung in which data are not normally distributed, tested by Mann Whitney. In graphs, each symbol represents a mouse, the horizontal line shows the, median and interquartile range for lung and lymph node data and mean and SEM error bars for the spleen data.

Values in B are included in the Source Data file.

Supplementary Figure 13: Lung structural cells can present IAV antigens to CD4 and CD8 T cells

A.

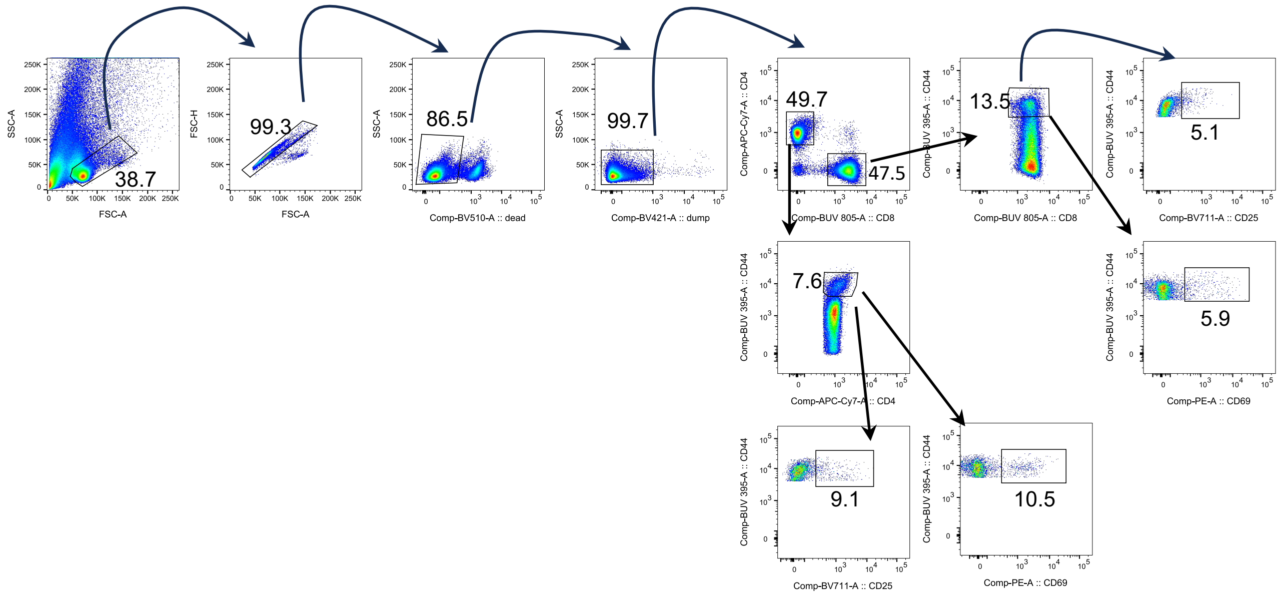

B.

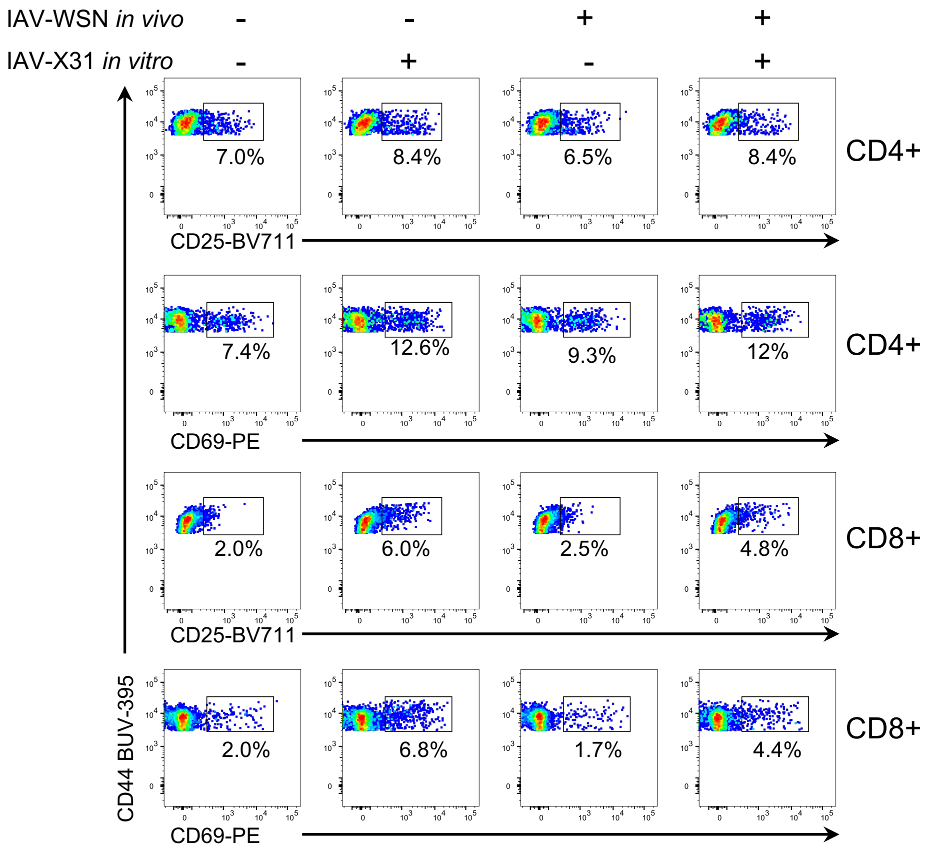

Supplementary Figure 13: Lung structural cells can present IAV antigens to CD4 and CD8 T cells

Lung CD45 negative cells were isolated from naïve or C57BL/6 mice infected 30 days earlier with IAV-WSN. After 24 hours, the cells were infected with IAV-X31 and co-cultured with T cells isolated from the spleens of mice infected with IAV-X31 9 days earlier. Activated CD4 and CD8 T cells were gated as shown in A and representative FACS plots from each group are shown in B. Data are representative of two experiments with 4 mice within each naïve and Day 30 IAV group per experiment.

Supplementary Figure 14: Lung structural cells only activate T cells through cognate antigen presentation

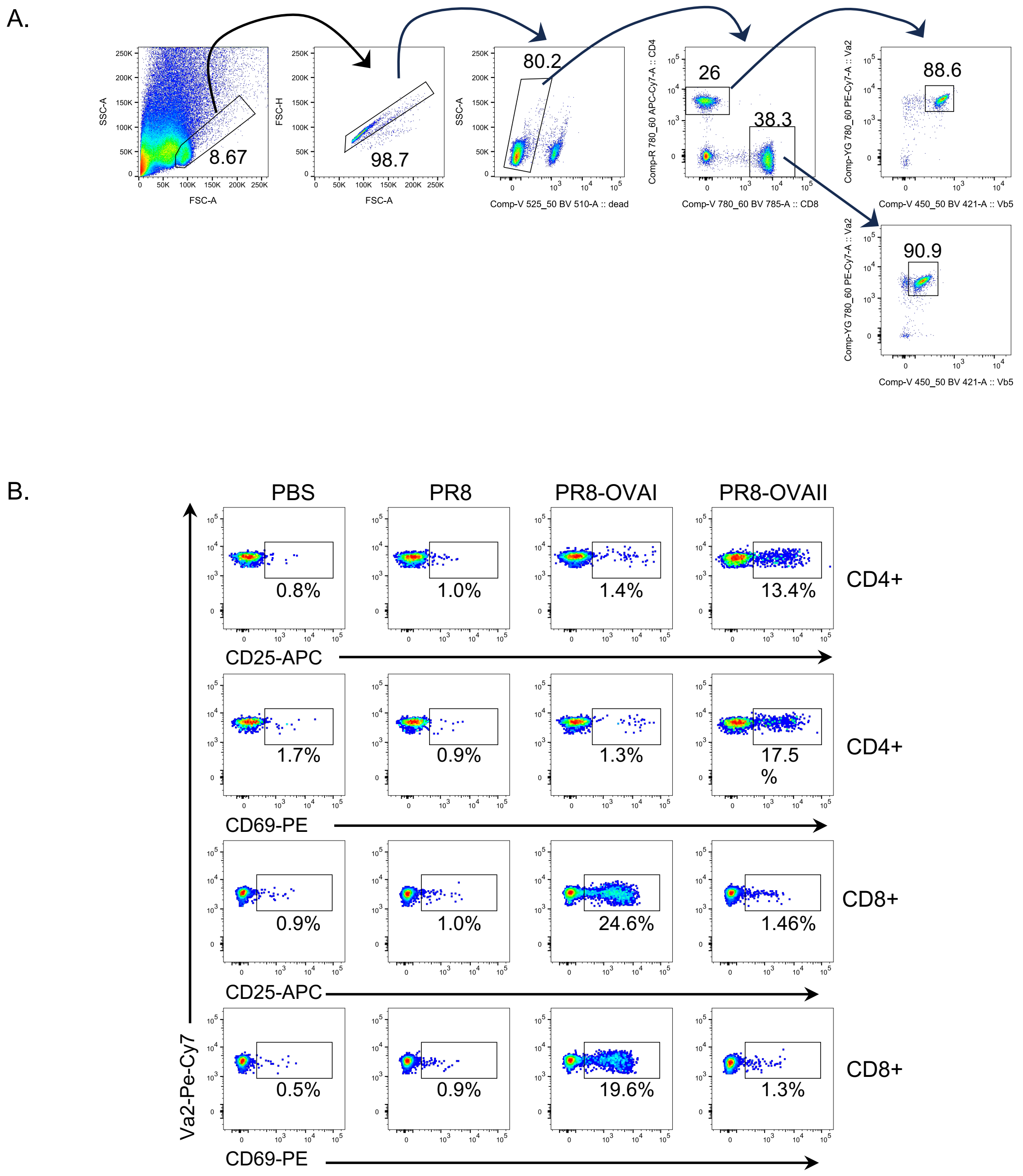

C.

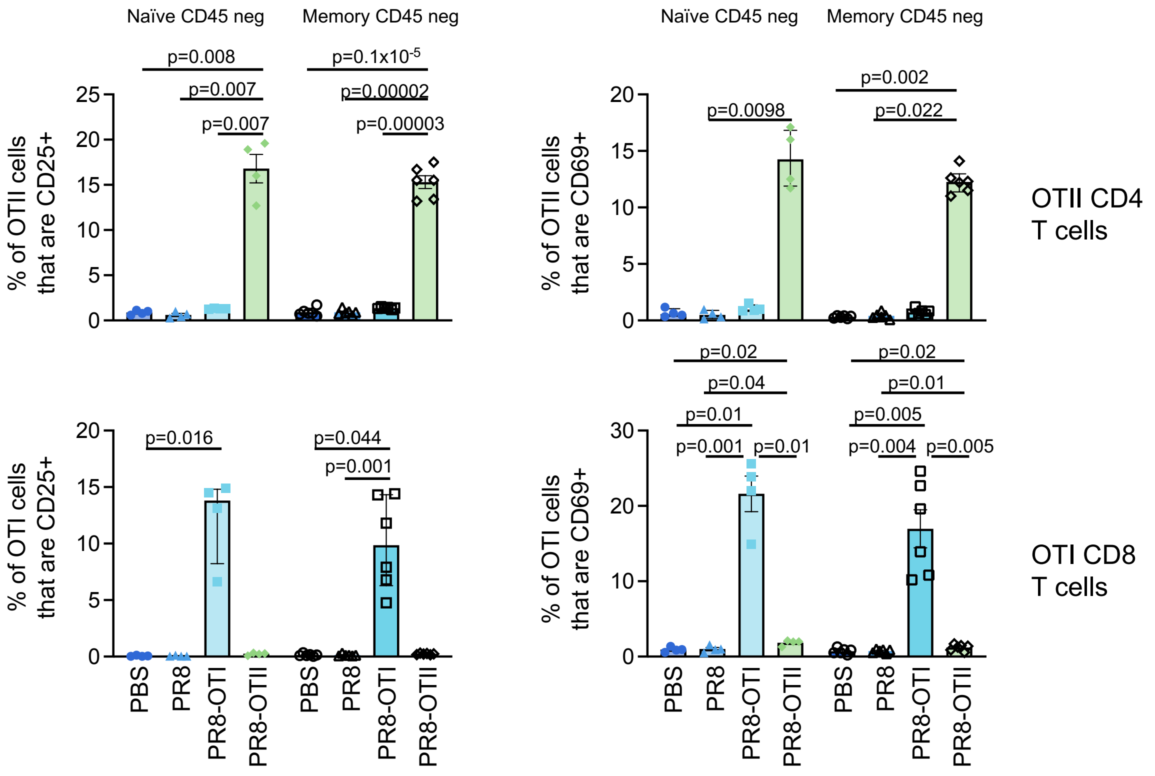

Supplementary Figure 14 Lung structural cells only activate T cells through cognate antigen presentation

Lung CD45 negative cells were isolated from naïve or C57BL/6 mice infected 73 days earlier with IAV-X31. After 24 hours, the cells were infected with IAV-PR8 as indicated and OTI and OTII cells added after a further 24 hours. T cell activation was examined after a further three days. OTI and OTII CD8 and CD4 T cells were gated as shown in A and representative FACS plots from each group shown in B and the data graphed in C.

Data are from one experiments with 4 mice within the naïve and 5 mice within the memory group. Data assessing OTII CD25 and OTI CD69 expression are normally distributed and data assessing OTII CD69 and OTI CD25 expression are not normally distributed. Comparisons between matched groups were tested by ANOVA followed by a Tukey's multiple comparison for normally distributed data or a Friedman's test followed by a Dunn's multiple comparison for data that were not normally distributed. Data between groups (naïve and memory CD45 negative cells) were tested by ANOVA and a Šidák's multiple comparison test or a Kruskal-Wallis test followed by Dunn's multiple comparison test for normal and not normally distributed data respectively. In graphs, each symbol represents a mouse, the horizontal line shows the mean and error bars are SEM for normally distributed data and the median and interquartile ranges are shown for graphs with data that are not normally distributed.

Values in C are included in the Source Data file.
