# Supplementary material for: Lung structural cells are altered by influenza virus leading to rapid immune protection following re-challenge
Source: Nat Commun. 2025 Aug 1;16:7061. doi: 10.1038/s41467-025-62364-y (PMC12317152; doi:10.1038/s41467-025-62364-y)
Supplement: Supplementary file 3 — Description of Additional Supplementary Files [file 41467_2025_62364_MOESM3_ESM.pdf]

Description of Additional Supplementary Files

File Name: Supplementary Data 1
Description: Transcript levels for differentially expressed genes in RNAseq experiment in Figure1

File Name: Supplementary Data 2
Description: Nanostring probe sequences and raw data from Nanostring experiment in Supplementary Figure 3

File Name: Supplementary Data 3
Description: SpiB target genes in lung epithelial cells and fibroblasts at day 10 and 40 post-influenza virus infection in Figure 3

File Name: Supplementary Data 4
Description: Gene expression comparison by nanostring primary versus re-infection in Figure 7

File Name: Supplementary Data 5
Description: Raw data from GeoMx experiment in Figure 7

File Name: Supplementary Data 6
Description: Sample information file for GeoMx experiment in Figure 7

File Name: Supplementary Data 7
Description: Analysed GeoMx data in Figure 7E and SFig 11
