# Supplementary material for: Lung structural cells are altered by influenza virus leading to rapid immune protection following re-challenge
Source: Nat Commun. 2025 Aug 1;16:7061. doi: 10.1038/s41467-025-62364-y (PMC12317152; doi:10.1038/s41467-025-62364-y)
Supplement: Supplementary file 11 — Reporting Summary [file 41467_2025_62364_MOESM11_ESM.pdf]

nature portfolio

Corresponding author(s): Megan MacLeod

Last updated by author(s): 7/5/2025

Reporting Summary

Nature Portfolio wishes to improve the reproducibility of the work that we publish. This form provides structure for consistency and transparency in reporting. For further information on Nature Portfolio policies, see our Editorial Policies and the Editorial Policy Checklist.

Statistics

For all statistical analyses, confirm that the following items are present in the figure legend, table legend, main text, or Methods section.

n/a	Confirmed	
☐	☒	The exact sample size (*n*) for each experimental group/condition, given as a discrete number and unit of measurement
☐	☒	A statement on whether measurements were taken from distinct samples or whether the same sample was measured repeatedly
☐	☒	The statistical test(s) used AND whether they are one- or two-sided<br>*Only common tests should be described solely by name; describe more complex techniques in the Methods section.*
☒	☐	A description of all covariates tested
☐	☒	A description of any assumptions or corrections, such as tests of normality and adjustment for multiple comparisons
☐	☒	A full description of the statistical parameters including central tendency (e.g. means) or other basic estimates (e.g. regression coefficient) AND variation (e.g. standard deviation) or associated estimates of uncertainty (e.g. confidence intervals)
☐	☒	For null hypothesis testing, the test statistic (e.g. *F*, *t*, *r*) with confidence intervals, effect sizes, degrees of freedom and *P* value noted<br>*Give P values as exact values whenever suitable.*
☒	☐	For Bayesian analysis, information on the choice of priors and Markov chain Monte Carlo settings
☒	☐	For hierarchical and complex designs, identification of the appropriate level for tests and full reporting of outcomes
☐	☒	Estimates of effect sizes (e.g. Cohen's *d*, Pearson's *r*), indicating how they were calculated

Our web collection on statistics for biologists contains articles on many of the points above.

Software and code

Policy information about availability of computer code

Data collection

Flow cytometry was collected and cells sorted using on BD FACSDIVA software (BD Biosciences, Versions 8 and 9), FACS ARIA IIU (sorting) and BDFortessa (analysis)
qPCR data collected via QuantStudio 7 flex
nCounter data collected via nCounter FLEX system, NanoString Technologies
RNA sequencing: Illumina NextSeq™ 500 platform
GeoMx: GeoMx Digital Spatial Profiler
Immunofluorescent slides: Images acquired on a LSM880 (Zeiss, Germany) using ZEN Black (Version 2.3).
H&E and RNAscope images acquired on: Aperio ImageScope version 12.1.0.5029, Aperio Technologies Inc., Vista
Cytokine data: Milliplex MCYTOMAG-70K assay and Milliplex MTH17MAG-47K-03 assay, acquired on a Luminx 200, R&D.

Data analysis

qPCR data: QuantStudio 7 flex.
nCounter Nanostring: NanoString nSolver analysis software, version 4.0
RNA-sequencing: STAR version 2.4.2a, FASTQC, FastP: Hisat2 and FeatureCounts. DESeq2 and FDR with a p-value of less than 0.05 was considered statistically significant. Further sample quality control, visualization and exploration were performed in R via SearchLight2. Over Representation Analysis and Network analysis: STRING11.5
GeoMx: Nanostring GeoMX DSP analysis suite Version 3
Normalised GeoMx data was analysed using R base functions and packages including Searchlight2
Immunofluorescent images: ZEN Blue (Version 2.3).
H&E and RNAscope: Aperio V9 algorithm, Aperio ScanScope XT System; Aperio Technologies.
Cytokine: Luminex software, R&D

Flow Data collection: BD FACSDiva Software version 9 (BD BioSciences)
Flow Data Analysis: FlowJo software Version 10 (BD)

For manuscripts utilizing custom algorithms or software that are central to the research but not yet described in published literature, software must be made available to editors and reviewers. We strongly encourage code deposition in a community repository (e.g. GitHub). See the Nature Portfolio guidelines for submitting code & software for further information.

Data

Policy information about availability of data

All manuscripts must include a data availability statement. This statement should provide the following information, where applicable:

- Accession codes, unique identifiers, or web links for publicly available datasets
- A description of any restrictions on data availability
- For clinical datasets or third party data, please ensure that the statement adheres to our policy

RNAseq data are available via GEO GSE278132, Nanostring and GeoMx data are included in Supplementary data excel and csv files, other primary data available on request to the corresponding authors.

Research involving human participants, their data, or biological material

Policy information about studies with human participants or human data. See also policy information about sex, gender (identity/presentation), and sexual orientation and race, ethnicity and racism.

Reporting on sex and gender	NA
Reporting on race, ethnicity, or other socially relevant groupings	NA
Population characteristics	NA
Recruitment	NA
Ethics oversight	NA

Note that full information on the approval of the study protocol must also be provided in the manuscript.

Field-specific reporting

Please select the one below that is the best fit for your research. If you are not sure, read the appropriate sections before making your selection.

☒ Life sciences ☐ Behavioural & social sciences ☐ Ecological, evolutionary & environmental sciences

For a reference copy of the document with all sections, see nature.com/documents/nr-reporting-summary-flat.pdf

Life sciences study design

All studies must disclose on these points even when the disclosure is negative.

Sample size	No statistical methods were used to predetermine the sample size. Samples sizes were based on similar studies from the last 20 years or for sequencing studies on a combination of experience, practical and expense limitations.
Data exclusions	In the RNAseq, one sample from the naïve endothelial cells and one from the day 40 fibroblasts were excluded due to poor quality. One sample in the flow data in Fig8D was excluded due to a technical error this sample could not be acquired.
Replication	Most experiments were repeated twice in independent experiments. The exceptions are the high cost sequencing based experiments and RNAscope studies. All attempts are replication were successful.
Randomization	No formal randomisation was used. Mice were housed in the same room and rack in the animal facility. Mice were sex- and age-matched within each experiments. In the anti-CD4/CD8 depletion studies, groups were mixed between cages to prevent cage-specific effects acting as a confounder.
Blinding	Researchers who counted plaques in the viral plaque assay and scored immune cell infiltrate in the H&E slides were blinded. The investigators were not blinded during collection of animal tissues due to requirements for cage identification and labeling for treatment purposes.

Reporting for specific materials, systems and methods

We require information from authors about some types of materials, experimental systems and methods used in many studies. Here, indicate whether each material, system or method listed is relevant to your study. If you are not sure if a list item applies to your research, read the appropriate section before selecting a response.

Materials & experimental systems

n/a	Involved in the study
☐	☒ Antibodies
☒	☐ Eukaryotic cell lines
☒	☐ Palaeontology and archaeology
☐	☒ Animals and other organisms
☒	☐ Clinical data
☒	☐ Dual use research of concern
☒	☐ Plants

Methods

n/a	Involved in the study
☒	☐ ChIP-seq
☐	☒ Flow cytometry
☒	☐ MRI-based neuroimaging

Antibodies

Antibodies used

Anti-CD45 FITC (BD, 30-F11), cat#:553080 at 1:200
anti-CD45-PE (BD, 30-F.11), cat#: 553081 at 1:200
anti CD45 eflour450 (ThermoFisher, 30-F.11), cat#:48-0451-82 at 1:100
anti-CD45 AF488 (ThermoFisher, clone:30-F11) cat#:53-0451-82 (1ug, i.v for 3 minutes)
anti-CD31 PE (Biolegend, MEC13.3), cat#:102508 at 1:400
anti-CD31-FITC (BioLegend, MEC13.3), cat#:102506 at 1:400
anti-CD326/EpCAM1-1 PerCP-Cy5.5 (Biolegend, G8.8), cat#:11219 at 1:200
anti-CD326/EpCAM1-1 BV711 (Biolegend, G8.8), cat#:118233 at 1:200
anti-CD140a/Pdgfra APC (Biolegend, APA5), cat#:135908 at 1:200
anti-CD140a/Pdgfra BV605 (Biolegend, APA5), cat#:135916 at 1:200
anti-Gp38/podoplanin PeCy7 (Biolegend, 8.8.1), cat#:127412 at 1:200
anti-Sca1 APC-Cy7 (BioLegend, W18174A), cat#:108126 at 1:200
anti-CD24 BV421 (Biolegend, M1/69), cat#:101825 at 1:100
anti-MHCII BUV395 (BD: 2G9), cat#:569244 at 1:200
anti-Siglec F APC (BioLegend, S17007L), cat#:155507 at 1:400
anti-CD11b-PeCy7 (BioLegend, M1/70), cat#:101216 at 1:200
anti-Ly6G-BV785 (BioLegend, 1A8), cat#:127645 at 1:200
anti-Ly6C-PerCP-Cy5.5 (ThermoFisher, HK1.4), cat#:128011 at 1:200
anti CD64 BV711 (BioLegend, X54-5/7.1), cat#:139311 at 1:200
anti-CD11c-eFluor780 (ThermoFisher, N418), cat#:47-0114-80 at 1:400
anti-B220 eFluor 450 (RA3 6B2, ThermoFisher), cat#:48-0452-82 at 1:400
anti-CD103-BV605 (BioLegend, 2E7), cat#:121433 at 1:200
anti-Bst2-PerCp-C5 (BioLegend, 927), cat#:127021 at 1:100
anti-CD9-PE (BioLegend, MZ3), cat#:124805 at 1:100
anti-CD4 APC-Alexa647 (RM4-5, ThermoFisher), cat#:557681 at 1:200
anti-CD8 BUV805 (BD 53-6.7), cat#: 564920 at 1:200
anti-CD44 BUV395 (BD, IM7), cat#740215 at 1:200
anti-CD25 BV711 (Biolegend, PC61), cat#:102049 At 1:200
anti-CD69 PE (BD, H1.2F3), cat#: 553237 at 1:200
anti-B220-PE ThermoFisher, RA3-6B2), cat#:12-0452-82 at 1:200
anti-MHCII eFluor 450 (M5114), cat#:48-5321-82 at 1:200
anti-F480 eFluor 450 (BM8), cat#:48-4801-82 at 1:200
anti-Ter119-FITC (BioLegend, TER-119), cat#:116205 at 1:200
anti-IAV-NP FITC (Invitrogen, D67J) ,cat#:MA1-7322 at 1:50
anti-Va2-PeCy7 (BD, B20.1), cat#:560624 at 1:400
anti-Vb5.1/5.2eFluor450 (ThermoFisher, MR9-4), cat#:48-5796-82 at 1:400
anti-CD25-APC (ThermoFisher, PC61.5), cat#:17-0251-82 at 1:200
anti-CD8a-BV785 (BioLegend, 53-6.7), cat#:100750 at 1:400
anti-Kb-Alexa 488 (BioLegend, AF6-88.5)cat#:116510 at 1:100
anti-CD49e-APC (BioLegend, 5H10-27), cat#:142605 at 1:100

Antibodies used for in vivo depletion studies: InVivoMab control rat IgG (LTF-2) cat#: BE0090-50MG, inVivoPlus anti-mouse CD4 (GK1.5) cat#: BP0003-1-100MG and InVivoPlus anti-mouse CD8 (2.43)BP0061-100MG, all from BioXCell.

Validation

Commercial antibodies have been validated by manufactures and the statements can be found on the manufactures' websites. For the in vivo depletion study, we followed the method described in PMID: 28783666 and depletion tested for our study, see Supplementary Figure 12.

Animals and other research organisms

Policy information about studies involving animals; ARRIVE guidelines recommended for reporting animal research, and Sex and Gender in Research

Laboratory animals	Mice were 10-12 weeks of age at the start of the experiment. Mice were either wildtype or SpiB-mCherry reporter mice, created at MRC Harwell, UK.
Wild animals	No wild animals were used in this study.
Reporting on sex	Mice were age- and sex-matched, experiments.<br>Wild-type C57BL/6NHsd mice were female mice, SpiB-mCherry mice were male and female mice, no formal sex specific analysis was performed and sex was considered a blocking factor in experimental design.
Field-collected samples	NA
Ethics oversight	Animal experiments were performed in accordance with home office regulations (Project Licenses P2F28B003 and PP1902420) and approved by the University of Glasgow Animal Welfare Ethics Review committee

Note that full information on the approval of the study protocol must also be provided in the manuscript.

Plants

Seed stocks	NA
Novel plant genotypes	NA
Authentication	NA

Flow Cytometry

Plots

Confirm that:

- [x] The axis labels state the marker and fluorochrome used (e.g. CD4-FITC).
- [x] The axis scales are clearly visible. Include numbers along axes only for bottom left plot of group (a 'group' is an analysis of identical markers).
- [x] All plots are contour plots with outliers or pseudocolor plots.
- [x] A numerical value for number of cells or percentage (with statistics) is provided.

Methodology

Sample preparation	For flow cytometry analysis of structural cells, single cell suspensions of lungs were prepared by enzymatic digestion with a final concentration of 1.6 mg/mL Dispase, 0.2 mg/mL collagenase P (Roche, UK) and 0.1 mg/mL DNase (Sigma, UK) for 40 minutes at 37°C in a shaking incubator and tissues disrupted by passing through a 100µm filter. Red blood cells were lysed with lysis buffer (ThermoFisher).<br><br>For analysis of CD4 and CD8 T cells by flow cytometry prior to euthanasia by cervical dislocation, mice were injected intravenously with 1µg AF488 conjugated anti-CD45 (clone, 30F11, ThermoFisher) and organs harvested after 3 minutes. Single-cell suspensions of lungs were prepared by digestion of snipped lung tissue with 1 mg/mL collagenase and 30µg/mL DNase (Sigma) for 40 minutes at 37 °C in a shaking incubator and tissues disrupted by passing through a 100µm filter. Spleens and lymph nodes were processed by mechanical disruption. Red blood cells were lysed from spleen and lungs with lysis buffer (ThermoFisher). Cells were counted using a hemocytometer with dead cells excluded using Trypan Blue.<br><br>Flow Cytometry Staining: Cells were incubated for 10 mins with Fc block (homemade containing 24G2 supernatant and mouse serum) surface stained with anti-CD45 FITC (Biolegend, 30-F11) or anti-CD45¬ PE (BD, 30-F.11) or anti CD45 eflour450 (ThermoFisher, 30-F.11), anti CD31 PE (Biolegend, MEC13.3) or anti-CD31-FITC (BioLegend, MEC13.3), CD326/EpCAM1-1 PerCP-Cy5.5 (Biolegend, G8.8) or CD326/EpCAM1-1 BV711 (Biolegend, G8.8), anti-CD140a/Pdgfra APC (Biolegend, APA5) or anti-CD140a/Pdgfra BV605 (Biolegend, APA5), Gp38/podoplanin PeCy7 (Biolegend, 8.8.1), anti-Sca1 APC-Cy7 (BioLegend, W18174A), anti-CD24 BV421 (Biolegend, M1/69), anti MHCII BUV395 (BD: 2G9), anti-Siglec F APC (BioLegend, S17007L), anti-CD11b-PeCy7 (BioLegend, M1/70), anti-Ly6G-BV785 (BioLegend, 1A8), anti-Ly6C-PerCP-Cy5.5 (ThermoFisher, HK1.4), anti CD64 BV711 (BioLegend, X54-5/7.1), anti-CD11c-eFluor780 (ThermoFisher, N418), anti B220 eFluor 450 (RA3 6B2,

ThermoFisher), anti-CD102-BV605 (BioLegend, 2E7), anti-Bst2-PerCp-C5 (BioLegend, 927) and anti-CD9-PE (BioLegend, MZ3), anti-Kb-Alexa 488 (BioLegend, AF6-88.5). Cells were stained with a fixable viability dye eFluor 780 or eFluor 506 (both ThermoFisher) as per the manufacturer's recommendations. Cells were fixed with cytofix/cytoperm (BD Bioscience) when required for 20 min at 4°C and stained in permwash buffer with anti-IAV-NP FITC (Invitrogen, D67J) for 1 h at room temperature.

For T cells, surface stains were anti-CD4 APC-Alexa647 (RM4-5, ThermoFisher), anti-CD8 BUV805 (BD 53-6.7), anti-CD44 BUV395 (BD, IM7), anti-CD25 BV711 (Biolegend, PC61), anti-CD69 PE (BD, H1.2F3) anti-B220 eFluor 450 (RA3-6B2), anti-MHCII eFluor 450 (M5114) and anti-F480 eFluor 450 (BM8) all ThermoFisher. Anti-B220, MHCII and F480 were used as a 'dump' gate. Samples were acquired on a BD Fortessa and analyzed using FlowJo (version 10 BD Bioscience, USA).
Additional Ab for TCR Tg studies: were Va2-PeCy7 (BD, B20.1), Vb5.1/5.2eFluor450 (ThermoFisher, MR9-4), CD25-APC (ThermoFisher, PC61.5) and CD8-BV785 (BioLegend, 53-6.7).

Lung digestion and isolation of mouse lung structural cells by Fluorescence-Activated Cell sorting: For FACS isolation of lung structural cells, single cell suspensions were generated by digesting snipped lungs with a final concentration of 3.2mg/mL Dispase-II, 0.4mg/mL Collagenase P, and 0.2mg/mL Dnase-1. Lung samples were incubated at 37°C for 20 minutes. Red blood cells were lysed using RBC buffer (ThermoFisher). Cells were simultaneously stained with CD45 microbeads (Miltenyi Biotec) and antibodies: anti-CD45 FITC (Biolegend, clone:30-F11) anti-Ter119-FITC (BioLegend, TER-119), anti-CD31 PE (BioLegend, MEC13.3), CD326/Epcam-1 PerCP Cy5.5 (Biolegend, G8.8), CD140a/Pdgfra APC (Biolegend, APA5). Hematopoietic cells were depleted using the LS MACS (Miltenyi Biotec) column as per manufacturer's instructions. CD45 depleted lung cells were stained with eFlour 780 viability stain (Thermofisher), and cells sorted on BD FACS Aria IIU sorter.

Instrument

BD LSR Fortessa (analysis), BD ARIA IIU (sorting)

Software

Data collection: BD FACSDiva Software version 9 (BD BioSciences)
Data Analysis: FlowJo software Version 10 (BD)

Cell population abundance

On each sort day, cells were collected, washed and re-run through the sorted to assess purity. These are reported in Supplementary Figure 1: representative purity: Epithelial cells: 97%, Fibroblasts: 94.5%, Endothelial cells: 97.5%.

Gating strategy

Supplementary Figure 1: Lung structural cells for FACS sorting and Main Figure 7.
Epithelial cells: FSC-A used to exclude debris, and viability dye used to exclude dead cells, FSC-A and FSC-H used to exclude doublets, CD45 and Ter119 used to exclude CD45+ and red blood cells, EpCAM-1+ CD140a negative.
Fibroblasts: FSC-A used to exclude debris, and viability dye used to exclude dead cells, FSC-A and FSC-H used to exclude doublets, CD45 and Ter119 used to exclude CD45+ and red blood cells, CD140a+, EpCAM-1 negative.
Blood endothelial cells: FSC-A used to exclude debris, and viability dye used to exclude dead cells, FSC-A and FSC-H used to exclude doublets, CD45 and Ter119 used to exclude CD45+ and red blood cells, CD140a negative, EpCAM-1 negative, CD31+ gp38 (podoplanin) negative.

Supplementary Figure 7B: SpiB-mCherry positive B cells and plasmacytoid DCs
B cells: FSC-A and SSC-A: used to include 'lymphocytes', FSC-A and FSC-H used to exclude doublets, Viability dye used to exclude dead cells, CD19+ and B220+ to include B cells, SpiB-m-Cherry.

Plasmacytoid DCs: FSC-A and SSC-A: used to include 'lymphocytes', FSC-A and FSC-H used to exclude doublets, Viability dye used to exclude dead cells, CD19negative, B220+, MHCII+, SigH+ and Spib-m-Cherry+.

Supplementary Figure 7C and SFig 8 SpiB-mCherry epithelial cells. Same gating used for Main Figures 4 and 5 up to EpCAM1+ cells.

SpiB+ ciliated cells: FSC-A used to exclude debris, and viability dye used to exclude dead cells, FSC-A and FSC-H used to exclude doublets, dump negative to exclude CD45 and CD31+, EpCAM1+, CD140a negative cells, Spib-mCherry+, CD24high Sca1+.
SpiB negative ciliated cells: FSC-A used to exclude debris, and viability dye used to exclude dead cells, FSC-A and FSC-H used to exclude doublets, dump negative to exclude CD45 and CD31+, EpCAM1+, CD140a negative cells, Spib-mCherry negative, CD24high Sca1+.
SpiB+ Club cells: FSC-A used to exclude debris, and viability dye used to exclude dead cells, FSC-A and FSC-H used to exclude doublets, dump negative to exclude CD45 and CD31+, EpCAM1+, CD140a negative cells, Spib-mCherry+, CD24-mid Sca1+.
SpiB negative club cells: FSC-A used to exclude debris, and viability dye used to exclude dead cells, FSC-A and FSC-H used to exclude doublets, dump negative to exclude CD45 and CD31+, EpCAM1+, CD140a negative cells, Spib-mCherry negative, CD24-mid Sca1+.
SpiB+ CD24+ progenitor cells: FSC-A used to exclude debris, and viability dye used to exclude dead cells, FSC-A and FSC-H used to exclude doublets, dump negative to exclude CD45 and CD31+, EpCAM1+, CD140a negative cells, Spib-mCherry+, CD24+ Sca1 negative.
SpiB negative CD24+ progenitor cells: FSC-A used to exclude debris, and viability dye used to exclude dead cells, FSC-A and FSC-H used to exclude doublets, dump negative to exclude CD45 and CD31+, EpCAM1+, CD140a negative cells, Spib-mCherry negatice, CD24+ Sca1 negative.
SpiB+ Sca1+ progenitor cells: FSC-A used to exclude debris, and viability dye used to exclude dead cells, FSC-A and FSC-H used to exclude doublets, dump negative to exclude CD45 and CD31+, EpCAM1+, CD140a negative cells, Spib-mCherry+, CD24 negative Sca1+.
SpiB negative CD24+ progenitor cells: FSC-A used to exclude debris, and viability dye used to exclude dead cells, FSC-A and FSC-H used to exclude doublets, dump negative to exclude CD45 and CD31+, EpCAM1+, CD140a negative cells, Spib-mCherry negatice, CD24negative Sca1+.

Supplementary Figure 9: Interferon responsive fibroblasts
FSC-A used to exclude debris, and viability dye used to exclude dead cells, FSC-A and FSC-H used to exclude doublets, CD140a + and dump negative to exclude CD45, CD31 and EPCAM1+ cells, CD49e+ CD9+, Bst2+.

Supplementary Figure 10A: Lung CD45+ Myeloid and B cells
Alveolar macrophages: FSC-A and SSC-A: large cell gate, FSC-A and FSA-H to exclude doublets, Viability dye negative to gate on live cells, CD45+ cells, SiglecF+ CD11b low.
Eosinophils: FSC-A and SSC-A: large cell gate, FSC-A and FSA-H to exclude doublets, Viability dye negative to gate on live cells, CD45+ cells, SiglecF-mid CD11b+
Neutrophils: FSC-A and SSC-A: large cell gate, FSC-A and FSA-H to exclude doublets, Viability dye negative to gate on live cells, CD45+ cells, SiglecF negative, CD11b+ Ly6G+.
MHCII low monocytes: FSC-A and SSC-A: large cell gate, FSC-A and FSA-H to exclude doublets, Viability dye negative to gate on live cells, CD45+ cells, SiglecF negative, CD11b+, Ly6C+, MHCII low.
MHCII low monocytes: FSC-A and SSC-A: large cell gate, FSC-A and FSA-H to exclude doublets, Viability dye negative to gate on live cells, CD45+ cells, SiglecF negative, Ly6G negative, CD11b+, Ly6C+, MHCII high.
CD11c+ macrophages: FSC-A and SSC-A: large cell gate, FSC-A and FSA-H to exclude doublets, Viability dye negative to gate on live cells, CD45+ cells, SiglecF negative, Ly6G negative, NOT Ly6ch/CD11bhi, CD11b+ CD64+, CD11c+ MHCII+.
Other macrophages: FSC-A and SSC-A: large cell gate, FSC-A and FSA-H to exclude doublets, Viability dye negative to gate on live cells, CD45+ cells, SiglecF negative, Ly6G negative, NOT Ly6ch/CD11bhi, CD11b+ CD64+, CD11c negative.
B cells: FSC-A and SSC-A: large cell gate, FSC-A and FSA-H to exclude doublets, Viability dye negative to gate on live cells, CD45+ cells, SiglecF negative, Ly6G negative, NOT Ly6ch/CD11bhi, CD64 negative, MHCII+ B220+, small cells within a lymphocyte gate.
Conventional DC1: FSC-A and SSC-A: large cell gate, FSC-A and FSA-H to exclude doublets, Viability dye negative to gate on live cells, CD45+ cells, SiglecF negative, Ly6G negative, NOT Ly6ch/CD11bhi, CD64 negative, B220 negative, CD11c+ MHCII+, CD103+ CD11b low.
Conventional DC2: FSC-A and SSC-A: large cell gate, FSC-A and FSA-H to exclude doublets, Viability dye negative to gate on live cells, CD45+ cells, SiglecF negative, Ly6G negative, NOT Ly6ch/CD11bhi, CD64 negative, B220 negative, CD11c+ MHCII+, CD103 negative CD11b+.
CD11b negative MHCII+: FSC-A and SSC-A: large cell gate, FSC-A and FSA-H to exclude doublets, Viability dye negative to gate on live cells, CD45+ cells, SiglecF negative, Ly6G negative, NOT Ly6ch/CD11bhi, CD64 negative, B220 negative, CD11c negative-low, CD11b negative, MHCII+.
CD11b+ MHCII low/negative: FSC-A and SSC-A: large cell gate, FSC-A and FSA-H to exclude doublets, Viability dye negative to gate on live cells, CD45+ cells, SiglecF negative, Ly6G negative, NOT Ly6ch/CD11bhi, CD64 negative, B220 negative, CD11c negative-low, CD11b+, MHCII-low-negative.

Supplementary Figure 12: CD4 and CD8 T cell depletion
CD4 T cells: FSC-A and SSC-A: lymphocyte gate, FSC-A and FSA-H to exclude doublets, Viability dye negative to gate on live cells, dump negative to remove B220+, MHCII+, and F4/80+ cells, CD4+ TCRb+.
CD8 T cells: FSC-A and SSC-A: lymphocyte gate, FSC-A and FSA-H to exclude doublets, Viability dye negative to gate on live cells, dump negative to remove B220+, MHCII+, and F4/80+ cells, CD8+ TCRb+.

Supplementary Figure 13: T cell gates for activated T cells
CD4 T cells: FSC-A and SSC-A: lymphocyte gate, FSC-A and FSA-H to exclude doublets, Viability dye negative to gate on live cells, dump negative to remove B220+, MHCII+, F4/80+ cells, CD4+ CD8 negative, CD44high, CD25 or CD69+.
CD8 T cells FSC-A and SSC-A: lymphocyte gate, FSC-A and FSA-H to exclude doublets, Viability dye negative to gate on live cells, dump negative to remove B220+, MHCII+, F4/80+ cells, CD8+ CD4 negative, CD44high, CD25 or CD69+.

Supplementary Figure 14: TCRTG cell gating:
OTI T cells:FSC-A and SSC-A: lymphocyte gate, FSC-A and FSA-H to exclude doublets, Viability dye negative to gate on live cells, CD8+ CD4negative cells, Va2+Vb+
OTII: SC-A and SSC-A: lymphocyte gate, FSC-A and FSA-H to exclude doublets, Viability dye negative to gate on live cells, CD4+ CD8negative cells, Va2+Vb5+.

☒ Tick this box to confirm that a figure exemplifying the gating strategy is provided in the Supplementary Information.
